# Amino Acid Derivatives as Palmitoylethanolamide Prodrugs: Synthesis, *In Vitro* Metabolism and *In Vivo* Plasma Profile in Rats

**DOI:** 10.1371/journal.pone.0128699

**Published:** 2015-06-08

**Authors:** Federica Vacondio, Michele Bassi, Claudia Silva, Riccardo Castelli, Caterina Carmi, Laura Scalvini, Alessio Lodola, Valentina Vivo, Lisa Flammini, Elisabetta Barocelli, Marco Mor, Silvia Rivara

**Affiliations:** Dipartimento di Farmacia, Università degli Studi di Parma, Parma, Italy; Sapienza University of Rome, ITALY

## Abstract

Palmitoylethanolamide (PEA) has antinflammatory and antinociceptive properties widely exploited in veterinary and human medicine, despite its poor pharmacokinetics. Looking for prodrugs that could progressively release PEA to maintain effective plasma concentrations, we prepared carbonates, esters and carbamates at the hydroxyl group of PEA. Chemical stability (pH 7.4) and stability in rat plasma and liver homogenate were evaluated by in vitro assays. Carbonates and carbamates resulted too labile and too resistant in plasma, respectively. Ester derivatives, prepared by conjugating PEA with various amino acids, allowed to modulate the kinetics of PEA release in plasma and stability in liver homogenate. L-Val-PEA, with suitable PEA release in plasma, and D-Val-PEA, with high resistance to hepatic degradation, were orally administered to rats and plasma levels of prodrugs and PEA were measured at different time points. Both prodrugs showed significant release of PEA, but provided lower plasma concentrations than those obtained with equimolar doses of PEA. Amino-acid esters of PEA are a promising class to develop prodrugs, even if they need further chemical optimization.

## Introduction

**Fig 1:**
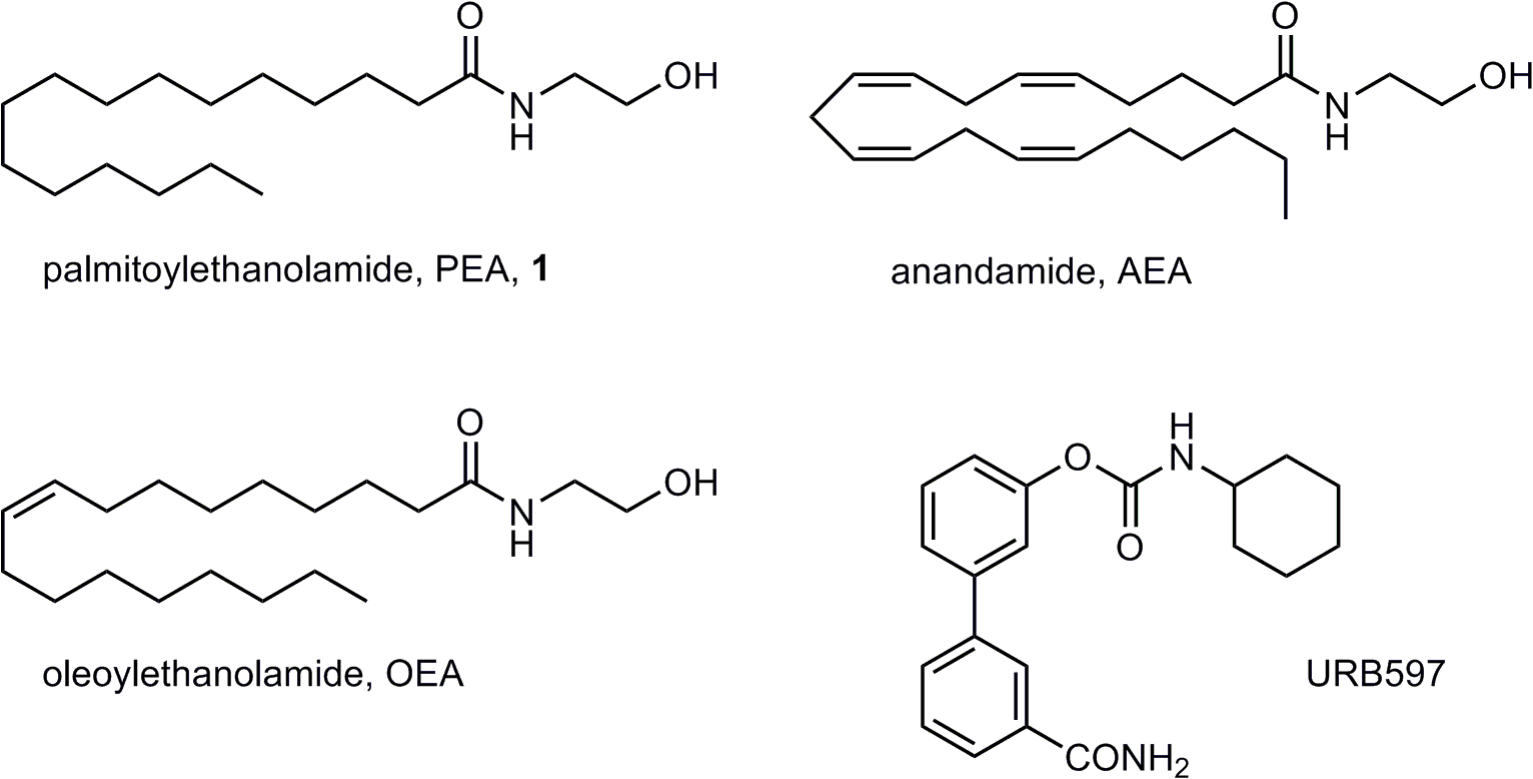
Fatty acid ethanolamides and FAAH inhibitor URB597.

Palmitoylethanolamide (PEA, **1**, Fig 1) is an endogenous lipid mediator belonging to the family of fatty acid ethanolamides (FAEs), which also includes the endocannabinoid *N*-arachidonoylethanolamine (anandamide, AEA, Fig 1) and the satiety factor oleoylethanolamide (OEA, Fig 1) [1]. FAEs are not stored in cells, but rather synthesized on demand from membrane phospholipid precursors, and their endogenous levels are regulated by enzymes responsible for their formation and degradation to fatty acids and ethanolamine. In particular, two enzymes are known to play a central role in the inactivating hydrolysis of FAEs: fatty acid amide hydrolase (FAAH) [2,3], an intracellular serine hydrolase belonging to the amidase signature (AS) family, and N-acylethanolamine acid amidase (NAAA) [4], a cysteine hydrolase localized in the lysosomes.

Regarding FAEs mechanism of action, while AEA acts as a full agonist of the cannabinoid receptor CB_1_ and as a partial agonist at CB_2_, the biological effects of OEA and PEA are mediated mainly *via* the activation of the peroxisome proliferator-activated receptor αPPAR-α) [5]. However, FAEs have multiple targets in different cells and tissues, with transient receptor potential vanilloid type 1 (TRPV1) [6], GPR129, GPR55 and other receptors also involved in their actions.

The role of PEA in inflammation and nociception has been largely documented. PEA has been shown to prevent mast cell activation [7] and reduce inflammatory pain *in vivo* in a variety of animal models [8,9,10]. Indeed, reduced PEA levels have been found in different inflammatory conditions, and PEA has been suggested to act as an endogenous signal able to prevent the development of acute inflammation [11,12]. A number of clinical trials have evaluated the effect of PEA on visceral, neuropathic and post-operative pain and PEA-containing preparations are approved by the European Community as dietary foods for special medical purposes and are commercially available for both veterinary and human use.

Despite its wide use, only a few data about the pharmacokinetics (PK) of exogenously administered PEA in humans or experimental animals are currently available. In humans, oral administration of PEA leads to a 2-9-fold increase in plasma baseline concentrations, depending on the dose [13]. In another recent study administration of 300 mg of ultramicronized PEA to healthy volunteers doubled plasma basal concentrations after 2 h, returning to basal levels after 4 h [14]. In animals, the time course of PEA plasma concentrations has been reported for Beagle dogs [15]. After oral administration of a 30 mg kg^-1^ dose, PEA reached the maximal plasma concentration (C_max_) 1–2 h after administration, with a five-fold increase in its basal plasma levels. Another PK profile of ultramicronized PEA after oral administration of a 15 mg kg^-1^ dose to Beagle dogs is reported in a US patent [16]. In this case, PEA reached the C_max_ 1 h after administration, with PEA basal concentrations only doubling and returning to basal values at t = 2 h. These data suggest that PEA, if orally administered at medium-high doses, produces limited systemic exposure levels, with plasma concentrations remaining in the nM range and with significant increases only for a short period of time.

In principle, different physicochemical and metabolic issues may be responsible for the limited exposure of oral PEA. The low aqueous solubility presumably limits PEA absorption, particularly at high doses. Moreover, in many tissues PEA is hydrolyzed to palmitic acid and ethanolamine by the enzymes NAAA and FAAH. In some cases, hydrolytic enzymes participate to the regulation of specific tissue levels of PEA, which play a role in the control of different processes, e.g. NAAA on inflammation [12]. Specific and non-specific amidases can also limit the oral bioavailability of exogenous PEA by a first-pass effect. In fact, liver is the second organ, after the brain, in which FAAH shows the highest specific activity [2].

PEA has recently received renewed interest by the scientific community and new approaches to increase PEA concentration *in vivo* have been attempted. Drug discovery efforts have focused on selective FAAH and NAAA inhibitors, which could restore physiological levels of PEA in those body districts in which it is down-regulated, for example during inflammation, effectively leading to anti-inflammatory and antinociceptive effects [12,17,18]. On the other hand, administration of exogenous PEA could be further exploited if its degradation in body fluids and tissues was reduced or controlled in some way. Thus, starting from the evaluation of PEA stability in rat plasma and liver, and from the assessment of the time course for its plasma levels after oral administration to Wistar rats, we explored the possibility to develop prodrugs of PEA by modification at its hydrolyzable alcohol group.

The prodrug approach has often been used to overcome problems caused by undesirable physicochemical and PK properties of a variety of drugs [19,20,21]. We applied this strategy to prepare PEA derivatives with the aim to prolong drug action upon oral administration, progressively releasing PEA and providing sustained plasma levels for a longer period of time compared to the direct administration of the drug. Indeed, a prodrug of PEA could facilitate an enhanced absorption and the circumvention of its metabolic inactivation in the liver. A prodrug of PEA having a galactosyl moiety, connected by an ester bond (through a succinyl spacer) to the hydroxyl group of PEA has been recently described [22]. This compound has been tested *in vitro* on cell cultures, showing improved cytoprotection in 6-hydroxydopamine-mediated cell death and prolonged intra-cellular levels of PEA.

In the present work, the hydroxyl group of PEA was selected as the functional group to be modified. Carbonate, ester and carbamate derivatives were prepared with the aim of testing their stability in biological tissues and of assessing their ability to increase PEA levels *in vivo*. In principle, the pro-moiety attached to the hydroxyl group should be cleaved by plasma esterases at a convenient rate to allow gradual and prolonged release of PEA to tissues. At the same time, the prodrug should be stable enough toward amidases to avoid degradation of the PEA moiety. The three functional groups linking PEA to the pro-moieties (carbonates, carbamates, esters) were selected to identify, at first, that/those having optimal stability in rat plasma and then to try chemical optimization of the pro-moiety.

**Fig 2:**
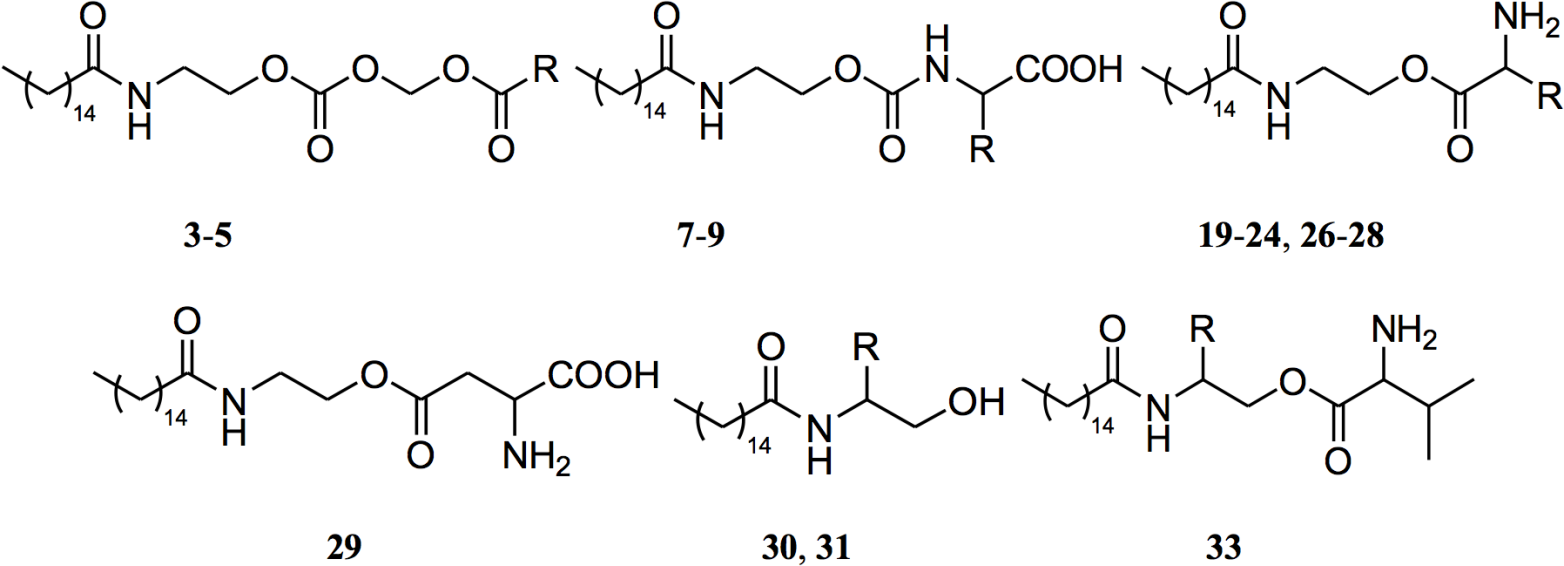
General formulas of PEA prodrugs reported in Table 1.

As carbonate prodrugs, we prepared acyloxymethylcarbonate derivatives (**3**–**5**, Fig 2 and Table 1) that are expected to be substrates of plasma esterases at the terminal acyloxy group. The size of this group was varied to test the possibility to modulate its plasma half-life.

**Table 1:** Stability data for palmitoylethanolamide (PEA, 1) and compounds 3–5, 7–9, 19–24, 26–31, 33.

			pH 7.4^a^	Rat Plasma^b^ ^,^ ^c^			Rat Liver^b^
Compd	Amino acid	R^d^	%Compd at 6 h	Compd *t*_1/2_ (min)	PEA *C*_max_ (nm)	PEA *t*_max_ (min)	Compd *t*_1/2_ (min)
**1**	-	-	95 ± 1	70 ± 2%^e^	72 ± 2	5	25 ± 5
**3**	-	Me	86 ± 1	<1	53 ± 5	5	<1
**4**	-	*i*-Pr	84 ± 1	1.1 ± 0.2	52 ± 2	5	<1
**5**	-	*c*-C_6_H_11_	84 ± 1	5.1 ± 0.5	52 ± 3	15	1.2 ± 0.2
**7**	L-Gly	H	101 ± 1	96 ± 4%^e^	-	-	23 ± 1
**8**	L-Ala	Me	103 ± 3	96 ± 2%^e^	-	-	29 ± 3
**9**	L-Val	*i*-Pr	99 ± 1	98 ± 1%^e^	-	-	92 ± 5%^f^
**19**	L-Ala	Me	64 ± 5	1.0 ± 0.1	65 ± 3	5	1.4 ± 0.5
**20**	L-Val	*i*-Pr	91 ± 2	46 ± 3	44 ± 4	300	26 ± 1
**21**	L-Ile	*s*-Bu	103 ± 6	205 ± 16	18 ± 1	360	65 ± 5%^f^
**22**	L-Trp	CH_2_-indol-3-yl	96 ± 1	4.5 ± 0.5	53 ± 1	30	36 ± 2
**23**	L-Asn	CH_2_CONH_2_	3.3 ± 0.3	9.0 ± 0.5	43 ± 4	60	24 ± 1
**24**	L-Gln	(CH_2_)_2_CONH_2_	61 ± 3	18 ± 1	22 ± 1	30	80 ± 10
**26**	α-L-Asp	CH_2_COOH	85 ± 4	172 ± 2	26 ± 5	240	66 ± 5%^f^
**29**	ω-L-Asp	-	83 ± 3	262 ± 35	34 ± 5	360	21 ± 1
**27**	D-Asn	CH_2_CONH_2_	3.5 ± 0.3	14 ± 1	36 ± 3	60	55 ± 4
**28**	D-Val	*i*-Pr	87 ± 2	173 ± 4	20 ± 1	360	67 ± 9%^f^
**30**		(*S*)-Me	96 ± 3	80 ± 4%^e^	-	-	79 ± 6%^f^
**31**		(*R*)-Me	95 ± 2	82 ± 4%^e^	-	-	87 ± 1%^f^
**33**	L-Val	(*R*)-Me	93 ± 4	190 ± 3	26 ± 1^g^	360^g^	38 ±± 2

^a^ Values expressed as percent remaining after 6 h of incubation at 37°C in PBS buffer pH 7.4.

^b^ *t*_1/2_ values calculated from pseudo first-order rate constants; mean ± SD, n = 3.

^c^ PEA basal level in 80% rat plasma = 20.1 ± 5.6 nM.

^d^ See Fig 2.

^e^ Percent remaining after 6 h incubation at 37°C in rat plasma.

^f^ Percent remaining after 2 h incubation at 37°C in rat liver homogenate.

^g^ Referred to (*R*)-α-methyl-PEA.

In the case of ester and carbamate prodrugs we prepared amino acid derivatives, attached to the linking group at their carboxyl or amino group, respectively. This pro-moiety was chosen to release, upon hydrolytic activation, two natural non-toxic compounds (i.e., PEA and the amino acid). In fact, conjugation with amino acids is a successful technique that has led to significant improvements in oral bioavailability when applied to a variety of drugs, such as the nucleoside analogs acyclovir and ganciclovir [23,24], and the anticancer drugs floxuridine [25] and gemcitabine [26]. While these prodrugs are esters at the amino acid carboxylic group, carbamates are another important class of prodrugs that had been exploited in several marketed compounds and offer the possibility to modulate their stability by structure-metabolism investigations [27]. We initially prepared carbamate and ester derivatives of PEA with L-glycine, L-alanine and L-valine (**7**–**9**, **19**, **20**) to evaluate the effect of side chain volume on prodrug stability and PEA release.

After preliminary results from the three series, a larger exploration was performed for ester derivatives, given their superior behavior (**21**–**24**, **26**–**29**). As steric bulk around a cleavable ester moiety is known to affect prodrug metabolism [28], we further derivatized PEA with hydrophobic amino acids endowed with branched (isoleucine, **21**) or planar (tryptophan, **22**) side chains. The hydrophilic/lipophilic balance of the prodrug is another physicochemical property that can impact enzymatic hydrolysis [29]. Hydrophilic amino acids with polar amide (asparagine, **23**; glutamine, **24**) or acidic (aspartate, **26**, **29**) side chains were therefore conjugated to PEA. Finally, we also tested the importance of amino acid chirality on PEA prodrug stability, synthesizing D-asparagine (**27**) and D-valine (**28**) ester derivatives. D-amino acid esters are usually characterized by a slower transformation rate to the parent compound compared to the corresponding L-amino acids in a biological setting, which could potentially lead to an improved tissue distribution [30,31].

The chemical stability of the newly synthesized prodrugs was evaluated in aqueous buffer at pH 7.4 and their bioconversion was assessed in rat plasma and rat liver homogenate. Analysis of *in vitro* results led to selection of candidates for the *in vivo* evaluation of their plasmatic concentration after oral administration to Wistar rats.

### Chemistry

**Fig 3:**
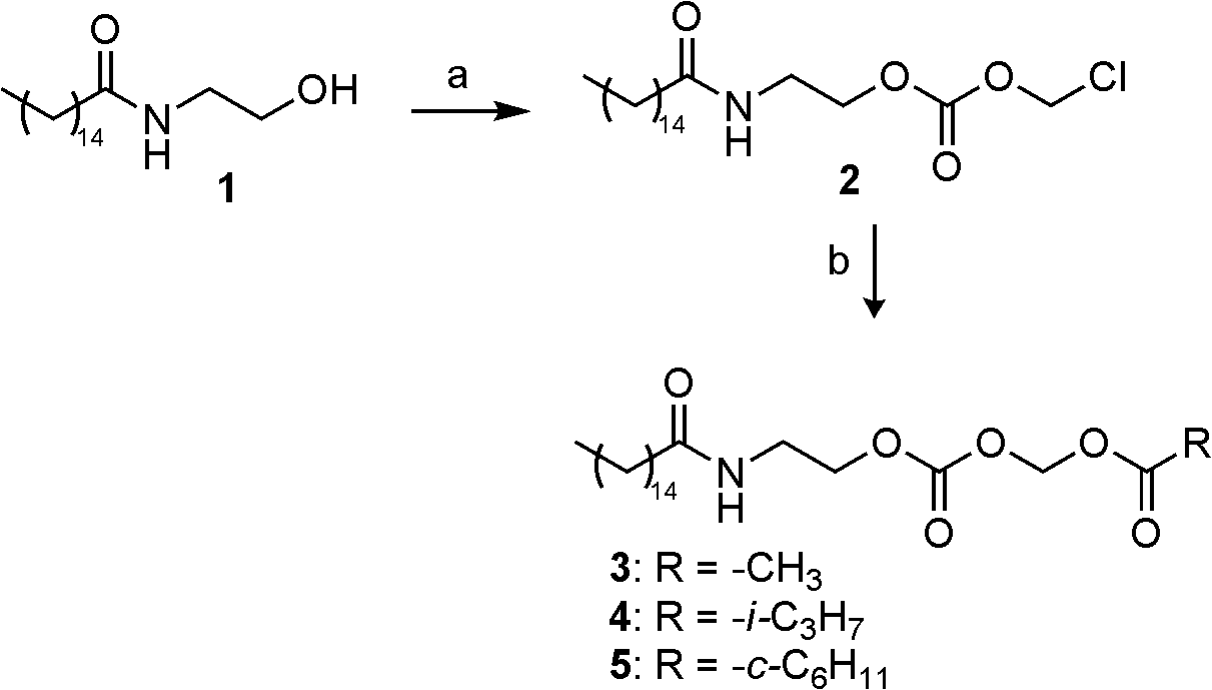
Synthesis of acyloxymethylcarbonates 3–5. Reagents and conditions: (a) chloromethyl chloroformate, pyridine, DCM, 0°C, 1 h; (b) RCOOCs, *tetra*-butyl ammonium iodide, acetonitrile, 80°C, 12 h.

**Fig 4:**
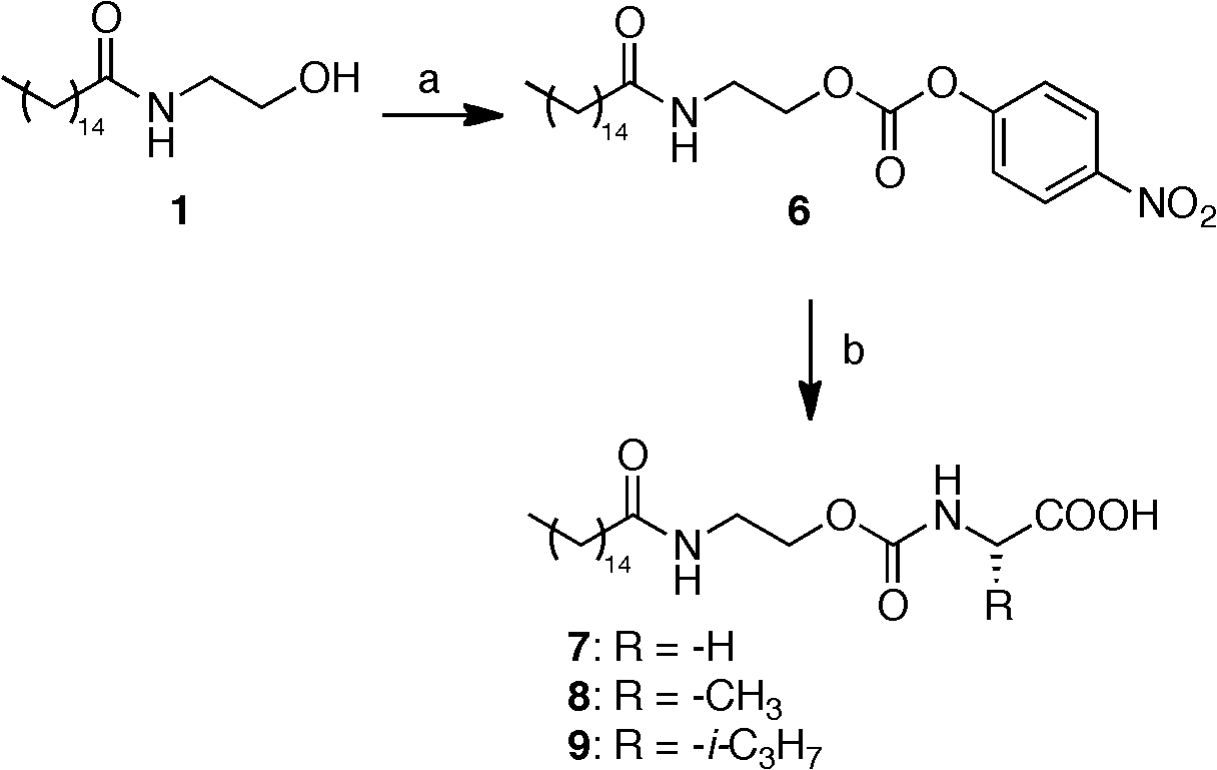
Synthesis of carbamates 7–9. Reagents and conditions: (a) p-nitrophenyl chloroformate, pyridine, DCM, 0°C to rt, 3 h; (b) RCH(NH_2_)COOH, Na_2_CO_3_, *tert*-butanol, water, 50°C, 12 h.

**Fig 5:**
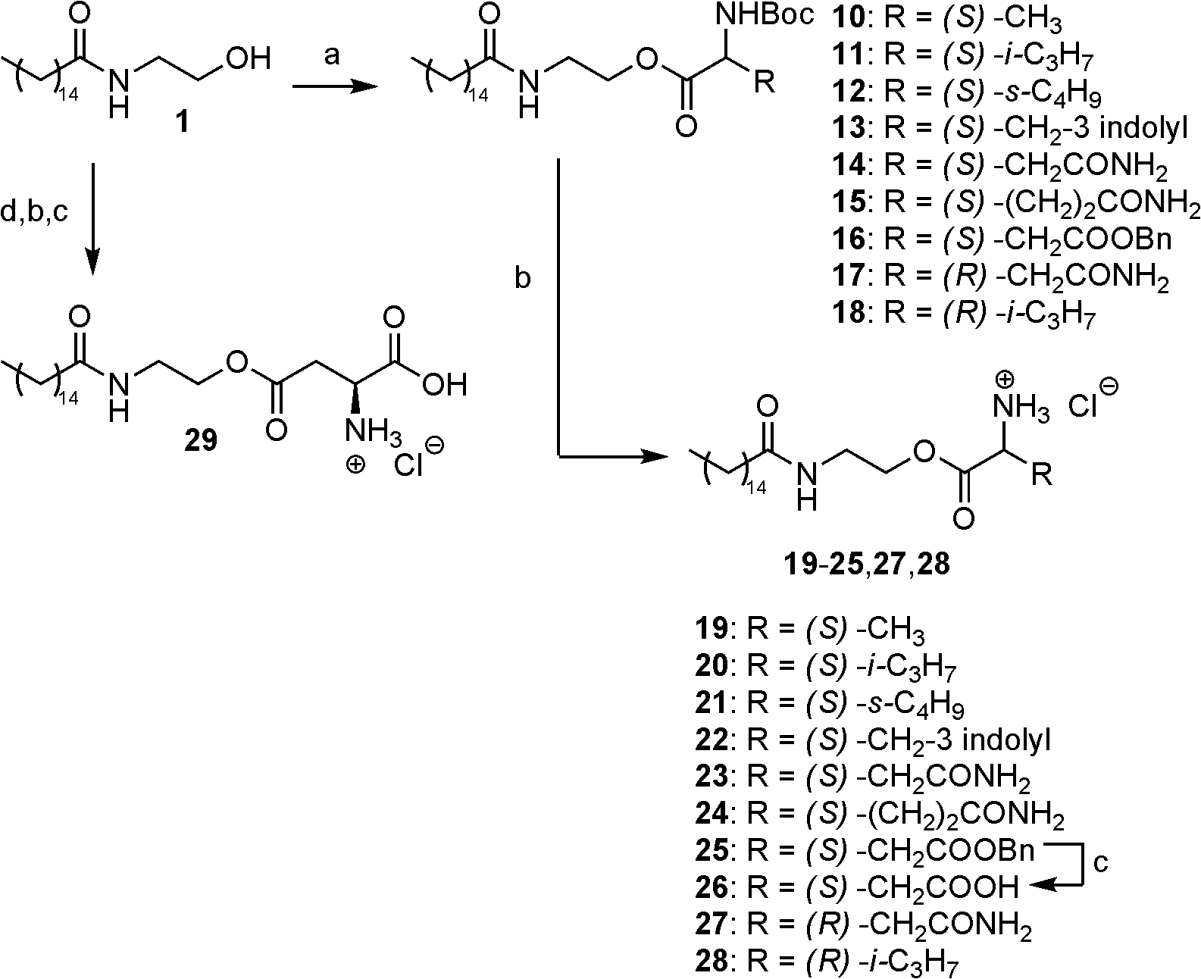
Synthesis of amino acid esters of PEA. Reagents and conditions: (a) RCH(NHBoc)COOH, DCC, DCM, 23°C, 12 h; (b) HCl, AcOEt, rt, or TFA/DCM 1:1 v/v, 0°C, 1–3 h, then HCl in AcOEt; (c) H_2_, Pd/C 10% w, HCl, EtOH, rt; (d) *N*-Boc-1-*O*-Bn-L- aspartic acid, DCC, DCM, 23°C, 12 h.

The synthesis of the three classes of prodrugs was conducted as outlined in Figs 3–5. Acyloxymethylcarbonates **3**–**5** (Fig 3) were obtained in modest yields *via* direct nucleophilic displacement of the chloride in **2** by the corresponding cesium carboxylate salts in boiling acetonitrile, employing tetrabutyl ammonium iodide as nucleophilic catalyst [32].

Analogously, carbamates **7**–**9** (Fig 4) were derived in good yields from the corresponding *p*-nitro phenol carbonate **6**, employing the corresponding amino acid. Sodium carbonate was used as base to set the amino group free, and the reaction was conducted in a mixture of *tert*-butanol and water to ensure homogeneity [33].

Ester prodrugs were accessed with a two-step sequence (Fig 5). Steglich esterification of the hydroxyl group of PEA with *tert*-butoxy carbonyl-protected amino acids was followed by acidic cleavage of such protecting group, either with hydrochloric acid in ethyl acetate, or by exposure to a chilled mixture of equal volumes of trifluoroacetic acid and dichloromethane. In the latter case, formation of the hydrochloride salt was achieved after a brief aqueous work-up, using a solution of HCl in methanol in a minimum amount of ethyl acetate. In the case of aspartate esters **26** and **29**, the additional carboxylate group was masked as benzyl ester and unveiled in the ultimate step by catalytic hydrogenolysis.

**Fig 6:**
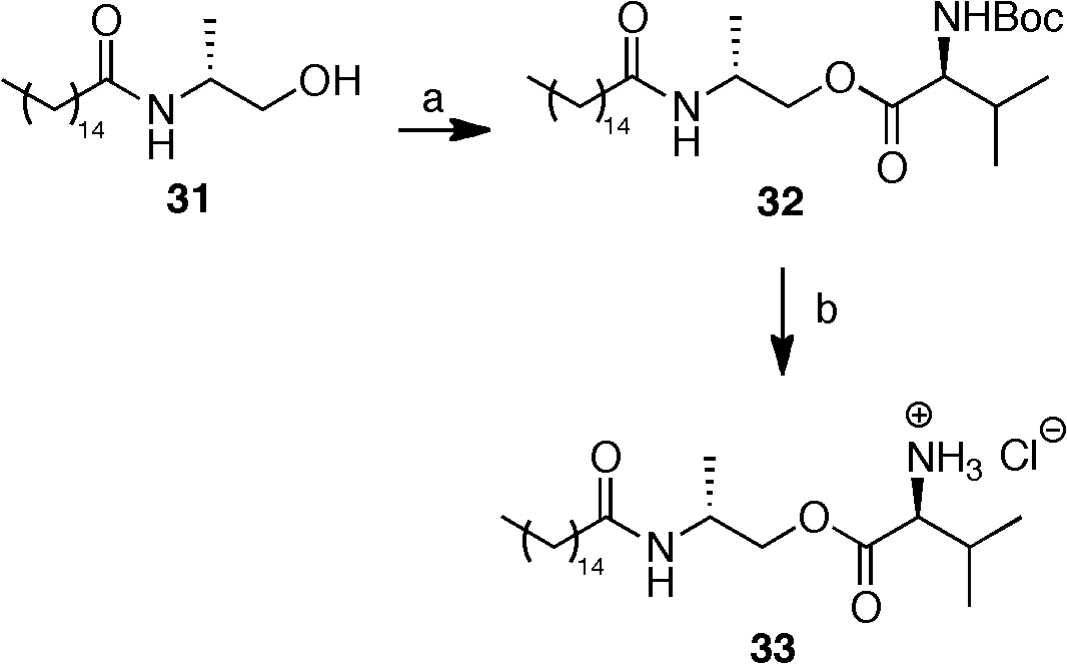
Synthesis of L-Val-(*R*)-α-methyl-PEA hydrochloride (33). Reagents and conditions: (a) *N*-Boc-L-Valine, DCC, DCM, 23°C, 12 h; (b) TFA/DCM 1:1 v/v, 0°C, 3 h then HCl, rt.

The same two-step procedure (esterification followed by acidic cleavage) was employed for the L-valine ester pro-drug **33** deriving from (*R*)- α-methyl PEA **31** (Fig 6), which in turn (along with its (*S*)-enantiomer **30**) was prepared as previously described [34].

## Materials and Methods

### Chemistry

Unless otherwise noted, reagents and solvents were purchased from commercial suppliers and were used without purification. The progress of the reactions was monitored by thin-layer chromatography with F254 silica-gel precoated sheets (Merck Darmstadt, Germany). UV light, ninhydrin ethanolic solution (0.3% w/v) and potassium permanganate solution (10% w/v) were used for detection. Flash chromatography was performed using Merck silica-gel 60 (Si 60, 40–63 μm, 230–400 mesh ASTM).

All reactions were carried out using flame-dried glassware under atmosphere of nitrogen. The ^1^H-NMR spectra were recorded on a Bruker Avance 400 spectrometer (400MHz); chemical shifts (δ scale) are reported in parts per million (ppm). ^1^H-NMR spectra are reported in the following order: multiplicity, number of protons and approximate coupling constants (J value); signals were characterized as s (singlet), d (doublet), t (triplet), quint. (quintuplet), m (multiplet), b (broad). Mass spectra were recorded on an Applied Biosystem API-150 EX system spectrometer with ESI or APCI interface. Purity of each final compound was assessed by HPLC-MS and found to be >95%.

HPLC-grade and analytical-grade solvents were purchased from Sigma-Aldrich (Milan, Italy). Water was freshly bidistilled and filtered before use. HPLC-ESI-MS-MS analyses were performed on a Thermo Accela UHPLC system equipped with an Accela OpenAS autosampler connected to a TSQ Quantum Access MAX triple quadrupole mass spectrometer (Thermo Italia, Milan, Italy) with an heated electrospray ionization (H-ESI) ion source. Data acquisition and processing were performed using Xcalibur software (version 2.1).

#### Chloromethyl 2-(palmitoylamino)ethyl carbonate (2)

To a chilled, stirred solution of PEA (**1**, 432.1 mg, 1.44 mmol) in anhydrous dichloromethane (DCM), pyridine (200 μL, 2.48 mmol) was added followed by chloromethyl chloroformate (150 μL, 1.69 mmol). The reaction proceeded for 1 h, when it was judged completed by TLC analysis. The reaction was quenched with water and diluted with AcOEt; the organic layer was washed with saturated sodium hydrogencarbonate, diluted HCl, water and brine. After drying over sodium sulfate, the solvents were removed under reduced pressure. The crude chloromethyl-carbonate ester was subjected to—silica gel column chromatography (*n*-hexane:AcOEt = 6:4) to furnish **2** (203.0 mg, 36%) as a white solid. ^1^H-NMR (CDCl_3_, 300 MHz) δ: 5.76 (bs, 3H), 4.34 (t, 2H, *J* = 5.1 Hz), 3.61 (q, 2H, *J* = 5.1), 2.21 (t, 2H, *J* = 7.4 Hz), 1.66–1.59 (bm, 2H), 1.35–1.20 (m, 24H), 0.90 (t, 3H, *J* = 6.4 Hz).

#### General procedure (A) for the synthesis of carbonates 3–5

Chloromethyl-carbonate ester **2** was dissolved in anhydrous acetonitrile. Tetrabutylammonium iodide (TBAI) (1.0 eq) was added at room temperature, followed by the cesium salt of the appropriate carboxylic acid (1.4 eq). The mixture was heated at 80°C with stirring under nitrogen atmosphere for 12 h. The reaction mixture was poured over water, diluted with AcOEt, and extracted two times with AcOEt. The combined organic layers were washed with water and then brine. The organic layer was dried over sodium sulfate and the solvents removed under reduced pressure. The crude product was subjected to flash column chromatography with an appropriate eluent mixture to furnish the purified product.

#### Acetoxymethyl 2-(palmitoylamino)ethyl carbonate (3)

Starting from cesium acetate (245.0 mg, 1.28 mmol) after silica gel column chromatography (*n*-hexane:AcOEt = 7:3), **3** (90.5 mg, 34%) was obtained as a white solid. ^1^H-NMR (CDCl_3_, 300 MHz) δ: 5.82–5.78 (m, 3H), 4.29 (t, 2H, *J* = 6.0 Hz), 3.59 (q, 2H, *J* = 6.0 Hz), 2.22–2.16 (m, 5H), 164 (m, 2H), 1.34–1.17 (m, 24H), 0.90 (t, 3H, *J* = 6.0 Hz). MS-APCI: Calc. for C_22_H_41_NO_6_: 415.3, found: 416.5 [M+H]^+^.

#### Isobutyroyloxymethyl 2-(palmitoylamino)ethyl carbonate (4)

Starting from cesium isobutyrate (200.3 mg, 0.91 mmol) after silica gel column chromatography (*n*-hexane:AcOEt = 7:3), **4** (14.2 mg, 7%) was obtained as a white solid. ^1^H-NMR (CDCl_3_, 400 MHz) δ: 5.84 (m, 1H), 5.76 (s, 2H), 4.26 (t, 2H, *J* = 5.6 Hz), 3.55 (q, 2H, *J* = 5.6 Hz), 2.61 (quintet, 1H, *J* = 7.0 Hz), 2.17 (t, 2H, *J* = 7.4 Hz), 1.27–1.17 (m, 30H), 0.87 (t, 3H, *J* = 6.6 Hz). MS-APCI: Calc. for C_24_H_45_NO_6_: 443.3, found: 444.5 [M+H]^+^.

#### (Cyclohexanelcarboxyloyloxy)methyl 2-(palmitoylamino)ethyl carbonate (5)

Starting from cesium cyclohexane-carboxylate (448.7 mg, 1.73 mmol) after silica gel column chromatography (*n*-hexane:AcOEt = 7:3), **5** (73.2 mg, 18%) was obtained as a white solid. ^1^H-NMR (CDCl_3_, 300 MHz) δ: 5.76 (m, 3H), 4.26 (t, 2H, *J* = 5.1 Hz), 3.56 (q, 2H, *J* = 5.1 Hz), 2.41–2.33 (m, 1H), 2.17 (t, 2H, *J* = 7.5 Hz), 1.94 (m, 2H), 1.77 (m, 2H), 1.74 (m, 2H), 1.61–1.58 (m, 2H), 1.52–1.39 (m, 2H), 1.25 (m, 24H), 0.90 (t, 3H, *J* = 6.6 Hz). MS-APCI: Calc. for C_27_H_49_NO_6_: 483.4, found: 484.6 [M+H]^+^.

#### 4-Nitrophenyl 2-(palmitoylamino)ethyl carbonate (6)

To a chilled, stirred solution of PEA (200.3 mg, 0.67 mmol) in anhydrous DCM, pyridine (100 μL, 1.24 mmol) was added followed by *p*-nitrophenyl chloroformate (269.1 mg, 1.34 mmol). The reaction was allowed to proceed while warming to room temperature over 3 h. The reaction was quenched pouring over water; the organic layer was washed with saturated sodium hydrogencarbonate, diluted aqueous HCl, water and brine. Following drying over sodium sulfate, the solvent was removed under reduced pressure. The crude *p*-nitrophenyl carbonate thus obtained was subjected to silica gel column chromatography (*n*-hexane:AcOEt = 2:1 to 1:1) to furnish **6** (186.5 mg, 60%) as a white solid. ^1^H-NMR (CDCl_3_, 300 MHz) δ: 8.31–8.26 (m, 2H), 7.41–7.36 (m, 2H), 5.84 (bt, 1H), 4.37 (t, 2H, *J* = 5.1 Hz), 3.65 (q, 2H, *J* = 5.1), 2.21 (t, 2H, *J* = 7.4 Hz), 1.68–1.59 (bm, 2H), 1.33–1.15 (m, 24H), 0.87 (t, 3H, *J* = 6.4 Hz). MS-APCI: Calc. for C_25_H_40_N_2_O_6_: 464.3, found: 465.5 [M+H]^+^.

#### General procedure (B) for the synthesis of compounds 7–9

4-Nitrophenyl carbonate **6** (1.4 eq) was mixed with the appropriate amino acid (1.0 eq) and Na_2_CO_3_ (4 eq). The obtained mixture was dissolved in a *tert*-butanol/water mix (40 mL, 3/2 v/v) and heated to 50°C for 24 h. The aqueous solution was then acidified with HCl to pH 2 and extracted with AcOEt. Following washing with brine, the organic layer was dried over sodium sulfate and the solvent removed under reduced pressure. The crude material was subjected to flash column chromatography with an appropriate eluent mixture to furnish the purified product.

#### N-(2-Palmitoylamino-ethyloxy)carbonyl-glycine (7)

Starting from glycine (27.8 mg, 0.37 mmol), after silica gel column chromatography (DCM:MeOH = 8:2 to 7:3) followed by trituration with diethylether, **7** (74.1 mg, 50%) was obtained as a white solid. ^1^H-NMR (DMSO-*d*_6_, 400 MHz) δ: 3.90 (t, 2H, *J* = 6.0 Hz), 2.17 (t, 2H, *J* = 7.4 Hz), 1.58 (m, 2 H), 1.33–1.15 (m, 24H), 0.88 (t, 3H, *J* = 6.4 Hz). MS-APCI: Calc. for C_21_H_40_N_2_O_5_: 400.3, found: 401.5 [M+H]^+^.

#### N-(2-Palmitoylamino-ethyloxy)carbonyl-L-alanine (8)

Starting from L-alanine (32.8 mg, 0.37 mmol), after silica gel column chromatography (DCM:MeOH = 97:3 to 9:1) followed by trituration with diethylether, **8** (142.6 mg, 93%) was obtained as a white solid. ^1^H-NMR (CDCl_3_/CD_3_OD/DMSO-*d*_6_, 400 MHz) δ: 3.92 (t, 2H, *J* = 5.7 Hz), 3.81 (q, 1H, *J* = 7.0 Hz), 3.25 (bt, 2H, *J* = 5.7 Hz), 2.05 (m, 1H), 1.47 (bm, 2H), 1.20 (m, 27H), 0.82 (t, 3H, *J* = 6.6 Hz). MS-APCI: Calc. for C_22_H_42_N_2_O_5_: 414.3, found: 437.5 [M+Na]^+^.

#### N-(2-Palmitoylamino-ethyloxy)carbonyl-L-valine (9)

Starting from L-valine (43.3 mg, 0.37 mmol), after silica gel column chromatography (DCM:MeOH = 97:3 to 9:1), **9** (148.9 mg, 91%) was obtained as a white solid. ^1^H-NMR (DMSO-*d*_6_, 400 MHz) δ: 8.10 (bs, 1H), 7.79 (bs, 1H), 3.92 (t, 2H, *J* = 5.7 Hz), 3.81 (q, 1H, *J* = 7.0 Hz), 3.25 (bt, 2H, *J* = 5.7 Hz), 2.05 (m, 2H), 1.47 (m, 2H), 1.21 (m, 24H), 0.84 (m, 9H). MS-APCI: Calc. for C_24_H_46_N_2_O_5_: 442.3, found: 465.5 [M+Na]^+^.

#### General procedure (C) for the synthesis of 2-(palmitoylamino)ethyl N-tert- butoxycarbonyl amino acid esters 10–18

PEA (1.0 eq) was dissolved in anhydrous DCM. *N*-Boc protected amino acid (1.2 eq) was added, followed by dicyclohexyl-carbodiimide (DCC, 1.2 eq). The obtained suspension was stirred at room temperature for 12 h. Precipitated dicyclohexyl-urea was removed filtering over a short pad of Celite. The clear filtrate was diluted with AcOEt and washed with water, aqueous sodium hydrogen carbonate and brine. The organic layer was dried over sodium sulfate and the solvents removed under reduced pressure. The crude was subjected to flash column chromatography with an appropriate eluent mixture to furnish the purified product.

#### 2-(Palmitoylamino)ethyl N-tert- butoxycarbonyl-L-alaninate (10)

Starting with *N*-Boc-L-alanine, silica gel column chromatography (*n*-hexane:AcOEt = 4:1) afforded **10** (84%) as a white solid. ^1^H-NMR (CDCl_3_, 300 MHz) δ: 6.11 (bs, 1H), 4.96 (bs, 1H), 4.25 (m, 3H), 3.54 (t, 2H, *J* = 6.0 Hz), 2.18 (t, 2H, *J* = 9.0 Hz), 1.65–1.52 (m, 2H), 1.45 (s, 9H), 1.38 (d, 3H, *J* = 9.0 Hz), 1.29–1.17 (m, 24H), 0.88 (t, 3H, *J* = 8.0 Hz). MS-APCI: Calc. for C_26_H_50_N_2_O_5_: 470.4, found: 471.5 [M+H]^+^.

#### 2-(Palmitoylamino)ethyl N-tert- butoxycarbonyl-L-valinate (11)

Starting with *N*-Boc-L-valine, silica gel column chromatography (*n*-hexane:AcOEt = 4:1) afforded **11** (95%) as a white solid. ^1^H-NMR (CDCl_3_, 300 MHz) δ: 6.07 (bs, 1H), 4.97 (bd, 1H), 4.30–4.15 (m, 2H), 4.08 (dd, 1H, *J* = 5.6, 8.0 Hz), 3.60–3.44 (m, 2H), 2.17 (t, 2H, *J* = 7.4 Hz), 2.07–2.14 (m, 1H), 1.61 (m, 2H), 1.44 (s, 9H), 1.29–1.17 (m, 24H), 0.98–0.85 (m, 9H). MS-APCI: Calc. for C_28_H_54_N_2_O_5_: 498.4, found: 499.5 [M+H]^+^.

#### 2-(Palmitoylamino)ethyl N-tert- butoxycarbonyl-L-isoleucinate (12)

Starting with *N*-Boc-L-isoleucine, silica gel column chromatography (*n*-hexane:AcOEt = 7:3) afforded **12** (81%) as a white solid. ^1^H-NMR (CDCl_3_, 400 MHz) δ: 6.05 (bs, 1H), 4.97 (bd, 1H), 4.28–4.11 (m, 3H), 3.60–3.44 (m, 2H), 2.17 (t, 2H, *J* = 7.6 Hz), 1.95–1.80 (m, 2H), 1.61 (m, 2H), 1.45 (s, 9H), 1.31–1.21 (m, 24H), 0.95–0.86 (m, 9H). MS-APCI: Calc. for C_29_H_56_N_2_O_5_: 512.4, found: 513.6 [M+H]^+^.

#### 2-(Palmitoylamino)ethyl N-tert- butoxycarbonyl-L-tryptophanate (13)

Starting with *N*-Boc-L-tryptophan, silica gel column chromatography (*n*-hexane:AcOEt = 7:3) afforded **13** (77%) as a white solid. ^1^H-NMR (CDCl_3_, 300 MHz) δ: 8.16 (s, 1H), 7.62 (d, 1H, *J* = 7.9 Hz), 7.40 (d, 1H *J* = 7.9 Hz), 7.20 (m, 2H), 7.08 (d, 1H, *J* = 2.2 Hz), 5.70 (bs, 1H), 5.09 (bd, 1H, *J* = 6.5 Hz), 4.59 (q, 1H, *J* = 6.7 Hz) 4.21–4.13 (m, 2H), 3.36–3.27 (m, 4H), 2.08–2.03 (m, 2H), 1.56 (m, 2H), 1.44 (s, 9H), 1.31–1.21 (m, 24H), 0.89 (t, 3H *J* = 6.3 Hz).

#### 2-(Palmitoylamino)ethyl N-tert- butoxycarbonyl-L-asparaginate (14)

Starting with *N*-Boc-L-asparagine, silica gel column chromatography (*n*-hexane:AcOEt = 8:2) afforded **14** (52%) as a white solid. ^1^H-NMR (CDCl_3_, 400 MHz) δ: 6.54 (bs, 1H), 5.83 (bs, 1H), 5.68 (bd, 1H), 5.37 (bs, 1H), 4.49–4.46 (m, 2H), 4.18–4.14 (m, 1H), 3.63–3.61 (m, 1H), 3.49–3.43 (m, 1H), 2.98 (dd, 1H, *J* = 4.0, 15.7 Hz), 2.80 (dd, 1H, *J* = 4.9, 15.7 Hz), 2.17 (t, 2H, *J* = 6.9 Hz), 1.63 (m, 2H), 1.45 (s, 9H), 1.31–1.21 (m, 24H), 0.88 (t, 3H, *J* = 6.7 Hz). MS-APCI: Calc. for C_27_H_51_N_3_O_6_: 513.4, found: 514.6 [M+H]^+^.

#### 2-(Palmitoylamino)ethyl N-tert- butoxycarbonyl-L-glutaminate (15)

Starting with *N*-Boc-L-glutamine, silica gel column chromatography (*n*-hexane:AcOEt = 8:2) afforded **15** (48%) as a white solid. ^1^H-NMR (CDCl_3_, 300 MHz) δ: 6.49 (bs, 1H), 6.31 (bs, 1H), 5.57–5.49 (bm), 4.36–4.28 (m, 2H), 4.22–4.17 (m, 1H), 3.58–3.54 (bm), 2.45–2.30 (m, 2H), 2.21 (t, 2H, *J* = 7.4 Hz)1.65–1.60 (m, 2H), 1.45 (s, 9H), 1.26 (m, 24H), 0.89 (t, 3H, *J* = 6.5 Hz).

#### 1-(2-(Palmitoylamino)ethyl) N-tert- butoxycarbonyl-O-benzyl-L-aspartate (16)

Starting with *N*-Boc-L-Asp—*O*-Bn, silica gel column chromatography (*n*-hexane:AcOEt = 7:3) afforded **16** (57%) as a white solid. ^1^H-NMR (CDCl_3_, 400 MHz) δ: 7.38–7.32 (m, 5H), 6.08 (bs, 1H), 5.43 (bd, 1H), 5.14 (m, 2H), 4.55 (m, 1H), 4.29–4.27 (m, 1H), 4.22–4.18 (m, 1H), 5.37 (bs, 1H), 4.49–4.46 (m, 2H), 4.18–4.14 (m, 1H), 3.63–3.61 (m, 1H), 3.49–3.43 (m, 1H), 3.07 (dd, 1H, *J* = 5.0, 17.0), 2.91 (dd, 1H, *J* = 5.0, 17.0), 2.15 (t, 2H, *J* = 7.2 Hz), 1.63 (m, 2H), 1.45 (s, 9H), 1.31–1.21 (m, 24H), 0.88 (t, 3H, *J* = 6.7 Hz). MS-APCI: Calc. for C_34_H_56_N_2_O_7_: 604.4, found: 605.4 [M+H]^+^.

#### 2-(Palmitoylamino)ethyl N-tert- butoxycarbonyl-D-asparaginate (17)

Starting with *N*-Boc-D-asparagine, silica gel column chromatography (*n*-hexane:AcOEt = 8:2) afforded **17** (66%) as a white solid. ^1^H-NMR (CDCl_3_, 400 MHz) δ: 6.54 (bs, 1H), 5.83 (bs, 1H), 5.68 (bd, 1H), 5.37 (bs, 1H), 4.49–4.46 (m, 2H), 4.18–4.14 (m, 1H), 3.63–3.61 (m, 1H), 3.49–3.43 (m, 1H), 2.98 (dd, 1H, *J* = 4.0, 15.7 Hz), 2.80 (dd, 1H, *J* = 4.9, 15.7 Hz), 2.17 (t, 2H, *J* = 6.9 Hz), 1.63 (m, 2H), 1.45 (s, 9H), 1.31–1.21 (m, 24H), 0.88 (t, 3H, *J* = 6.7 Hz). MS-APCI: Calc. for C_27_H_51_N_3_O_6_: 513.4, found: 514.6 [M+H]^+^.

#### 2-(Palmitoylamino)ethyl N-tert- butoxycarbonyl-D-valinate (18)

Starting with *N*-Boc-D-valine, silica gel column chromatography (*n*-hexane:AcOEt = 4:1) afforded **18** (89%) as a white solid. ^1^H-NMR (CDCl_3_, 300 MHz) δ: 6.07 (bs, 1H), 4.97 (bd, 1H), 4.30–4.15 (m, 2H), 4.08 (dd, 1H, *J* = 5.6, 8.0 Hz), 3.60–3.44 (m, 2H), 2.17 (t, 2H, *J* = 7.4 Hz), 2.07–2.14 (m, 1H), 1.61 (m, 2H), 1.44 (s, 9H), 1.29–1.17 (m, 24H), 0.98–0.85 (m, 9H). MS-APCI: Calc. for C_28_H_54_N_2_O_5_: 498.4, found: 499.5 [M+H]^+^.

#### General procedure (D) for the synthesis of 2-(palmitoylamino)ethyl amino acid esters from Boc-protected precursors. Synthesis of 2-(palmitoylamino)ethyl L-alaninate hydrochloride (19)

Compound **10** (124.7 mg, 0.251 mmol) was dissolved in 5 mL of anhydrous AcOEt. At 0°C, a room temperature-saturated solution of HCl in AcOEt was added dropwise. The mixture was kept at room temperature while being stirred for 16 h when the hydrochloride salt precipitated as a white powder. The solid was filtered and washed with few small portions of Et_2_O, to obtain **19** (91.0 mg, 91%), as a white solid. ^1^H-NMR (DMSO-*d*_6_, 400 MHz) δ: 8.49 (bs, 3H), 8.09 (bs, 1H), 4.15–4.11 (m, 2H), 2.07 (t, 2H, *J* = 7.2 Hz), 1.47 (m, 2 H), 1.40 (d, 3H, *J* = 7.0 Hz) 1.33–1.15 (m, 24H), 0.84 (t, 3H, *J* = 6.2 Hz). MS-APCI: Calc. for C_21_H_42_N_2_O_3_: 370.3, found: 371.6 [M+H]^+^.

#### 2-(Palmitoylamino)ethyl L-isoleucinate hydrochloride (21)

Following procedure D, compound **21** (212.9 mg, 71%) was obtained as a white powder. ^1^H-NMR (CD_3_OD, 300 MHz) δ: 4.31–4.26 (m, 2H), 3.93 (d, 1H, *J* = 4.0 Hz), 3.58–3.42 (m, 2H), 2.19 (t, 2H, *J* = 7.3), 1.59–1.48 (m, 2H), 1.28 (m, 24H), 1.03–0.96 (m, 6H), 0.90 (t, 3H, *J* = 6.9 Hz). MS-APCI: Calc. for C_24_H_48_N_2_O_3_: 412.4, found: 413.6 [M+H]^+^.

#### 2-(Palmitoylamino)ethyl L-tryptophanate hydrochloride (22)

Following procedure D, compound **22** (151.1 mg, 75%) was obtained as a white powder. ^1^H-NMR (CD_3_OD, 300 MHz) δ: 7.56 (m, 1H), 7.40 (m, 1H), 7.20 (s, 1H), 7.18–7.05 (m, 2H), 4.32 (dd, 1H, *J* = 5.3, 8.1 Hz), 4.28–4.24 (m, 2H), 3.53–3.36 (m, 3H), 2.17 (t, 2H, *J* = 7.2 Hz), 1.61–1.54 (m, 2H), 1.28–1.23 (m, 24H), 0.9 (t, 3H, *J* = 6.5 Hz). MS-APCI: Calc. for C_29_H_47_N_3_O_3_: 485.4, found: 486.6 [M+H]^+^.

#### 2-(Palmitoylamino)ethyl L-glutaminate hydrochloride (24)

Following procedure D, compound **24** (265.2 mg, 84%) was obtained as a white powder. ^1^H-NMR (DMSO-*d*_6_, 400 MHz) δ: 8.69 (bs, 3H), 8.19 (t, 1H, *J* = 5.6 Hz), 7.49 (bs, 1H), 6.9 (bs), 4.20–4.08 (m, 2H), 3.98 (t, 1H, *J* = 6.4 Hz). 2.35–2.18 (m, 2H), 2.08 (t, 2H, *J* = 9.1 Hz), 2.04–1.98 (m, 2H), 1.48–1.44 (m, 2H), 1.23 (m, 24H), 0.85 (t, 3H, *J* = 6.6 Hz). MS-APCI: Calc. for C_23_H_45_N_3_O_4_: 427.3, found: 428.5 [M+H^+^].

#### 1-(2-(Palmitoylamino)ethyl) O-benzyl-L-aspartate (25)

Following procedure D, compound **25** (153.4 mg, 78%) was obtained as a white powder. ^1^H-NMR (CD_3_OD, 300 MHz) δ: 7.37 (m, 5H), 5.23 (m, 2H), 4.42 (t, 1H, *J* = 5.1 Hz), 4.26 (t, 2H, *J* = 4.6 Hz), 3.45 (m, 2H), 3.13 (d, 2H, *J* = 5.0 Hz), 2.19 (t, 2H, J = 7.4 Hz), 1.60 (m, 2H), 1.30 (m, 24H), 0.92 (t, 3H, *J* = 6.3 Hz).

#### 2-(Palmitoylamino)ethyl D-asparaginate hydrochloride (27)

Following procedure D, compound **27** (135.2 mg, 82%) was obtained as a white powder. ^1^H-NMR (CD_3_OD, 300 MHz) δ: 4.28 (m, 3H), 3.53–3.41 (m, 2H), 2.96 (t, 2H *J* = 6.5 Hz), 2.19 (t, 2H, *J* = 7.3), 1.61–1.56 (m, 2H), 1.30–1.22 (m, 24H), 0.88 (t, 3H, *J* = 6.6 Hz). MS-APCI: Calc. for C_22_H_43_N_3_O_4_: 413.3, found: 414.6 [M+H^+^].

#### General procedure (E) for the synthesis of 2-(palmitoylamino)ethyl amino acid esters from Boc-protected precursors. Synthesis of 2-(palmitoylamino)ethyl L-valinate hydrochloride (20)

Compound **11** (363.0 mg, 0.728 mmol) was dissolved in 5 mL of anhydrous DCM, under nitrogen atmosphere. At 0°C, trifluoroacetic acid (TFA, 5 mL) was added and the reaction mixture was stirred and allowed to warm to room temperature over 3 h. When the starting material was no longer detected by TLC analysis, the mixture was poured over water and diluted with a mixture of AcOEt and Et_2_O (1:1 v/v, 100 mL). The organic layer was basified with a NaHCO_3_/Na_2_CO_3_ aqueous buffer until no more carbon dioxide was produced, and then washed with water and brine. After removal of the solvents under reduced pressure, the crude material was dissolved in AcOEt, and a solution of HCl in MeOH (c/a 1.25 M, 1 mL) was added at 0°C. The hydrochloride salt precipitated as a white powder. The solid was filtered and washed with few small portions of Et_2_O, to obtain **20** (260.4 mg, 82%) as a white solid. ^1^H-NMR (CD_3_COOD, 300 MHz) δ: 4.44–4.37 (m, 1H), 4.31–4.23 (m, 1H), 4.12 (d, 1H, *J* = 4.7 Hz), 3.68–3.47 (m, 2H), 2.42–2.29 (m, 3H), 1.62 (m, 2H), 1.30 (m, 24H), 1.12–1.08 (m, 6H), 0.89 (t, 3H, *J* = 6.2 Hz). MS-APCI: Calc. for C_23_H_46_N_2_O_3_: 398.4, found: 399.6 [M+H^+^].

#### 2-(Palmitoylamino)ethyl L-asparaginate hydrochloride (23)

Following procedure E, compound **23** (79.2 mg, 52%) was obtained as a white powder. ^1^H-NMR (CD_3_OD, 300 MHz) δ: 4.28 (m, 3H), 3.53–3.41 (m, 2H), 2.96 (t, 2H *J* = 6.5 Hz), 2.19 (t, 2H, *J* = 7.3), 1.61–1.56 (m, 2H), 1.30–1.22 (m, 24H), 0.88 (t, 3H, *J* = 6.6 Hz). MS-APCI: Calc. for C_22_H_43_N_3_O_4_: 413.3, found: 414.6 [M+H]^+^.

#### 2-(Palmitoylamino)ethyl D-valinate hydrochloride (28)

Following procedure E, compound **28** (260.4 mg, 82%) was obtained as a white solid. ^1^H-NMR (CD_3_COOD, 300 MHz) δ: 4.44–4.37 (m, 1H), 4.31–4.23 (m, 1H), 4.12 (d, 1H, *J* = 4.7 Hz), 3.68–3.47 (m, 2H), 2.42–2.29 (m, 3H), 1.62 (m, 2H), 1.30 (m, 24H), 1.12–1.08 (m, 6H), 0.89 (t, 3H, *J* = 6.2 Hz). MS-APCI: Calc. for C_23_H_46_N_2_O_3_: 398.4, found: 399.6 [M+H^+^].

#### 1-(2-(Palmitoylamino)ethyl) L-aspartate hydrochloride (26)

To a solution of **25** (143.2 mg, 0.28 mmol) in EtOH, HCl (1.25 M in MeOH, 220 μL) was added, followed by Pd/C (10% w). The mixture was evacuated and backfilled with hydrogen three times before being stirred under a slight overpressure (double jacketed balloon) of hydrogen for 12 h. After filtration over Celite, the solvents were removed under reduced pressure to give compound **26** (92.5 mg, 80%) as a white powder. ^1^H-NMR (DMSO-*d*_6_, 400 MHz) δ: 8.37 (bs, 3H), 8.04 (t, 1H, *J* = 5.4 Hz), 4.13 (m, 2H), 3.97 (t, 1H, *J* = 6.1 Hz), 2.36–2.18 (m, 2H), 2.09 (t, 2H, *J* = 7.2), 1.46 (m, 2H), 1.22 (m, 24H), 0.84 (t, 3H, *J* = 6.6 Hz). MS-APCI: Calc. for C_22_H_42_N_2_O_5_: 414.3, found: 415.5 [M+H]^+^.

#### 4-(2-(Palmitoylamino)ethyl) L-aspartate hydrochloride (29)

PEA (300.2 mg, 1.00 mmol) was dissolved in anhydrous DCM (40 mL). *N*-Boc-L-Asp-1-*O*-Bn (389.1 mg, 1.20 mmol) was added, followed by N,N-dimethyl-4-aminopyridine (DMAP, 36.7 mg, 0.30 mmol) and DCC (248.0 mg, 1.2 mmol). The obtained suspension was stirred at room temperature for 12 h. Precipitated urea was removed filtering over a short pad of Celite. The clear filtrate was diluted with AcOEt and washed with water, aqueous sodium hydrogen carbonate and finally brine. The organic layer was dried with sodium sulfate and the solvents removed under reduced pressure. The crude was subjected to flash column chromatography (*n*-hexane:AcOEt = 7:3) to furnish the intermediate fully protected compound as a transparent sticky syrup. Cleavage of the Boc group was performed following general procedure D, and the crude hydrochloride salt was dissolved in EtOH (30 mL). 1 M aqueous HCl (600 μL) was added, followed by Pd/C (10%, catalytic amount). The mixture was evacuated and back-filled with hydrogen for three times, then allowed to stir for 12 h under a slight overpressure (double jacketed balloon) of hydrogen. A small amount of Celite was added and the slurry thus obtained was filtered over a pad of Celite. The clear solution was evaporated under reduced pressure to afford a white powder (187 mg, 83%). ^1^H-NMR (DMSO-*d*_6_, 400 MHz) δ: 8.39 (bs, 3H), 7.95 (t, 1H, *J* = 5.4 Hz), 4.22 (t, 1H, *J* = 5.4 Hz), 4.05 (t, 2H, *J* = 5.4 Hz), 2.92 (d, 2H, *J* = 5.4 Hz). 2.06 (t, 2H, *J* = 8.9 Hz), 1.49–1.47 (m, 2H), 1.40–1.20 (m, 24H), 0.85 (t, 3H, *J* = 6.5 Hz). MS-APCI: Calc. for C_22_H_42_N_2_O_5_: 414.3, found: 415.5 [M+H^+^].

#### Synthesis of (2R)-2-(palmitoylamino)propyl N-benzyloxycarbonyl-L-valinate (32)

Following general procedure C, starting from **31**, compound **32** was obtained as a white solid after silica gel column chromatography (*n*-hexane:AcOEt = 7:3). ^1^H-NMR (CDCl_3_, 400 MHz) δ: 5.68 (d, 1H, *J* = 6.3 Hz), 4.97 (d, 1H, *J* = 7.3 Hz), 4.30–4.07 (m, 4H), 2.14 (t, 2H, *J* = 7.6 Hz), 1.60 (m, 2H), 1.45 (s, 9H), 1.31–1.21 (m, 24H), 1.18 (d, 3H, *J* = 6.6 Hz), 0.98 (d, 1H, *J* = 6.9 Hz), 0.92–0.86 (m, 6H). MS-APCI: calc. for C_29_H_56_N_2_O_5_: 512.4; found: 513.7 [M+H^+^].

#### Synthesis of (2R)-2-(palmitoylamino)propyl L-valinate hydrochloride (33)

Cleavage of the Boc group from **32** was performed following general procedure E, to furnish compound **33** as a white powder. ^1^H-NMR (CD_3_OD, 300 MHz) δ: 4.28–4.08 (m, 3H), 3.92 (d, 1H, *J* = 4.6 Hz), 2.33–2.25 (m, 1H), 2.17 (t, 2H, *J* = 6.6 Hz), 1.62–1.57 (m, 2H), 1.28 (bs, 24H), 1.18 (d, 3H, *J* = 6.6 Hz), 1.08–1.05 (m, 6H), 0.90 (t, 3H, *J* = 6.5 Hz). MS-APCI: calc. for C_24_H_48_N_2_O_3_: 412.4, found: 413.6 [M+H^+^], 435.5 [M+Na^+^].

#### Chemical and metabolic stability of PEA and of PEA prodrugs

The chemical and metabolic stability of PEA and all prodrugs were studied by incubating each compound (5 μL of a 5 μm solution in DMSO, or DMSO as control) with 495 μL of 10 mM phosphate buffered saline pH 7.4 for chemical stability, 80% v/v rat plasma or 20% w/v rat liver homogenate prepared accordingly to previously described procedures [35] for metabolic stability, reaching a final concentration of 50 nm for the test compound.

Rat plasma and liver samples were pre-incubated at 37°C for 10 min before adding the compounds. After incubation for different time periods (0, 15, 30, 60, 120, 240 and 360 min), samples were deproteinized by adding 100 μL of acetonitrile with 75 nmol L^-1^ IS (PEA-d_4_). The samples were centrifuged (14 000 g, 10 min, 4°C), and the supernatants were analyzed by HPLC–ESI–MS/MS. For each tested compound, a multiple reaction monitoring (MRM) ESI–MS/MS method was set up in order to monitor the relative concentration of compound and released PEA at the different incubation times, compared to the time t = 0, in rat plasma and liver homogenate.

C_max_ and t_max_ in rat plasma were read from the raw data as the coordinates of the highest concentration observed. A Thermo TSQ Quantum triple-quadrupole mass spectrometer (Thermo Italia, Milan, Italy) with heated electrospray ionization (H-ESI) ion source was used for mass detection and analysis. Mass spectrometric analyses were done in positive ion mode. H-ESI interface parameters were set as follows: probe middle (D) position; capillary temperature 270°C; spray voltage 3.0 kV. Nitrogen was used as nebulizing gas at the following pressure: sheath gas 35 psi; auxiliary gas 15 arbitrary units (a.u.). Argon was used as the collision gas at a pressure of approximately 1.5 mtorr (1 torr = 133.3 Pa). For quantitative analysis, the following parent ion → product ions transitions were selected: PEA (**1**): m/z 300.2 → m/z 62.3 (tube lens 60 V; collision energy 19 eV); PEA-d_4_ (Internal Standard): m/z 304.2 → m/z 66.3 + m/z 287.2 (tube lens 72 V; collision energy 15, 12 eV). MRM transitions, tube lens voltages and collision energies for compounds **3–5**, **7–9**, **19**–**24**, **26**–**31**, and **33** are reported in the S1 File. Standard curves for all the analytes, generated on three different working days, showed good linearity in the 1.0–2500 nmol mL^−1^ concentration range. The coefficients of correlation (r^2^) were >0.99 for all curves. The LOQ was 1 nmol mL^−1^ for all analytes. Extraction efficiency was determined by comparing the peak area ratio of spiked samples at three concentration levels (low, intermediate, and high) to those of extracted charcoal treated blank plasma spiked with the corresponding concentrations. The mean extraction recovery ranged from 109% to 91% for all compounds. The specificity of the assay was evaluated by comparison of HPLC–ESI–MS/MS chromatograms of the analytes at the LOQ to those of charcoal treated blank plasma samples in triplicate.

### Pharmacology

All experiments were carried out maintaining the animals in compliance with the Guide for the Care and Use of Laboratory Animals published by the US National Institutes of Health (NIH Publication No. 85–23, revised in 1996). Research protocols were performed according to ARRIVE guidelines. This study was approved by the local Ethical Committee for Animal Experimentation of the University of Parma and the Italian Ministry of Health (D. Lgs 26/2014 ex D.Lgs.116/92, authorization number 17/2014-B).

Twenty-seven male Wistar rats (Charles River Laboratories, Italy), 150–250 g in weight, were used in the study. Number of animals was calculated by G-power *a priori* analysis (α = 0.05 and β = 0.05). Animals were housed in group in policarbonate cages (Tecniplast, Italy) in a conventional facility with controlled temperature 22±1°C, a relative humidity of 55±5%, and a 12 h light/dark cycle (6:00 am to 6:00 pm). The rats were provided standard pellet diet (4RF21 GLP Mucedola, Italy) and water *ad libitum* throughout the study. The experiments were started only after acclimatization of animals to the laboratory conditions. Before the experiments, rats were fasted for 18 h, with free access to drinking water. Animals were randomized in three experimental groups (nine animals for each group) receiving respectively L-Val-PEA (**20**), D-Val-PEA (**28**) and PEA (**1**).

The day of the experiments substances were turned into a corn oil suspension, after ultrasound and vortexing and they were administered to rats between 8.00 and 10.00 am by gastric gavage at the equimolar doses of 145 mg kg^-1^ for L- and D-Val-PEA (**20, 28**) and 100 mg kg^-1^ of PEA (**1**). Blood samples were collected via sub-tongue puncture [36] from each anaesthetized rat (2% isofluran in air) to minimize any suffering. Blood samples of 0.2 mL were collected before treatment and at 15, 30, 60, 120, 240, 360 and 480 min post-dosing into EDTA-treated tubes (Sarstedt, Germany), immediately centrifuged (5000 rpm, 10 min, 4°C; refrigerated microcentrifuge Z 216 MK, Hermle LaborTechnik). Resulting plasma was immediately processed by protein precipitation with ice-cold acetonitrile containing the IS 75 nmol L^-1^ (PEA-d_4_) and analyzed as previously described. AUC values were calculated employing the software add-ons of Microsoft Excel [37].

### Docking studies

Chains A and B were extracted from the crystal structure of rat FAAH (PDB code 1MT5) [38] in its covalent adduct with methylarachidonyl phosphonate (MAP), and they were prepared using the Protein Preparation Wizard [39] available in Maestro 9.6 [40]. The bound inhibitor was removed and hydrogen atoms were added to the structure. Ionizable amino acids were modelled in their charged form, with the exception of the catalytic K142 which was maintained neutral. The tautomeric form of histidines was chosen to maximize the number of hydrogen bonds with neighboring residues.

The structure of compound **20** was built with standard Maestro tools and it was optimized with the OPLS2005 force field [41], using a convergence gradient of 0.05 kJ mol^-1^ Å^-1^. Ligand docking was performed with Glide 6.1 [42] in the standard precision mode into the active site of monomer B of FAAH, collecting fifty poses for subsequent analysis. Docking studies were performed into a grid centered on MAP with the inner and the outer boxes set to 24 and 44 Å, respectively. Hydrogen bond constraints were applied between the amide oxygen of **20** and the amide nitrogens of Ile238 and Gly239 backbone, and between the amide nitrogen of **20** and the backbone carbonyl of Met191. The best ranked complex according to the Emodel values was minimized using the OPLS2005 force field implemented in MacroModel 10.2 [43], applying the Polak-Ribiere conjugate gradient method to a convergence threshold of 0.05 kJ mol^-1^ Å^-1^. The ligand and residues within 8 Å from it were free to move, while backbone of the other residues was kept fixed.

## Results and Discussion

### *In vitro* assessment of prodrug stability

Prodrug stabilities were evaluated in phosphate buffered saline at pH 7.4, rat plasma and rat liver homogenate. Rat plasma includes a rich arsenal of esterases, such as carboxylesterases and cholinesterases [44], and gives an indication of the susceptibility of the newly synthesized prodrugs to hydrolytic activation *in vivo*. Rat liver homogenate was chosen as a model to predict first-pass metabolism of prodrugs. Beyond esterase activity, liver is also known to have the ability to catalyze the hydrolysis of fatty acid amides, which could result in degradation of the PEA component, either after release from the prodrug or at the prodrug stage itself. In fact, the specific amidase FAAH has high expression levels in the liver [2]. The results of chemical and enzymatic stability assays on PEA (**1**) and compounds **3**–**5**, **7**–**9**, **19**–**24**, **26**–**31** and **33** are reported in Table 1.

Chemical stability was evaluated by measuring, in buffer at physiological pH (pH 7.4), both the residual concentration of the starting compound and PEA release by mass spectrometry (HPLC-ESI-MS/MS). In these conditions PEA is very stable, with 95% of compound still present after 6 h. For acyloxymethylcarbonates **3**–**5** about 85% of compound was recovered after 6 h, while carbamates **7**–**9** remained completely intact. The stability of the ester-based prodrugs varied as a function of the amino acid side chain. A dependence of chemical stability on the size and shape of the amino acid side chain could be clearly observed: the branched L-isoleucine derivative **21** was more stable than the planar L-tryptophan **22** and the smaller L-valine **20** and L-alanine **19** conjugates. The L- (**23**) and D-asparagine (**27**) derivatives were not stable in these conditions, with only 3% of starting prodrug remaining unaltered after 6 h. While in general the amount of prodrug disappeared after 6 h was quantitatively transformed into PEA and amino acid, in the case of **23** and **27** a slow deamidation occurred at the amino acid side chain, producing about 1% of L- and D-aspartate derivatives, respectively (data not shown).

PEA (**1**) was rather stable when added to a solution composed by pooled rat plasma (80% v/v) and phosphate buffer pH 7.4. PEA had basal levels of 20.1±5.6 nM in the starting solution of rat plasma. After addition of 25 pmol of PEA in 500 αL, PEA levels showed a biphasic trend. From PEA concentrations of 71.9±2.1 nM at starting point, we observed an initial decay to 56.2±4.3 nM after 1 h, followed by a much slower decrease, with a value of 44.0±3.2 nM after 6 h (See Fig A in S2 File). On the other hand, FAAH is highly expressed in rat liver [2], where a number of other hydrolases are also present. In fact, we observed that PEA is quickly metabolized in rat liver homogenate, with a half-life of about 25 min, a PEA concentration of 4.0±1.2 nM after 120 min and no detectable basal PEA levels.

Prodrugs **3**–**5**, carrying the acyloxymethylcarbonate pro-moiety, exhibited very fast conversion to parent **1** in both rat plasma and liver homogenate (see Figs B-D in S2 File for time course of prodrugs hydrolysis in rat plasma and liver homogenate). The introduction of a bulky substituent, such as the cyclohexyl ring in **5**, provided a decrease in the rate of conversion to **1**, but this effect was marginal on compound stability, leading to a half-life of 5 min and a maximum concentration of PEA reached after 15 min. These compounds were even less stable in rat liver homogenate, with half-lives shorter than or equal to 1 min.

The subset of carbamate-based prodrugs **7**–**9** was very stable in rat plasma with only < 4% of prodrug hydrolyzed after a 6 h incubation. Their hydrolysis in rat liver was dependent on the steric hindrance of the side chain, with L-Val-PEA (**9**) displaying higher stability than L-Gly (**7**) and L-Ala (**8**) analogues, suggesting a role for specific recognition at esterase catalytic sites. We have already reported a shielding effect of a bulky alkyl chain on esterase-catalyzed hydrolysis for carbamates acting as prodrugs of imidazole H_3_-antagonists [45] and as selective FAAH inhibitors [46]. Even if it might be possible to further modulate the stability of these two classes of compounds, we decided to stop the preparation of carbamate- or carbonate-based PEA prodrugs as they did not display suitable stability properties to maximize drug exposure, being too stable in rat plasma (carbamates) or too labile in both biological matrices (carbonates).

**Fig 7:**
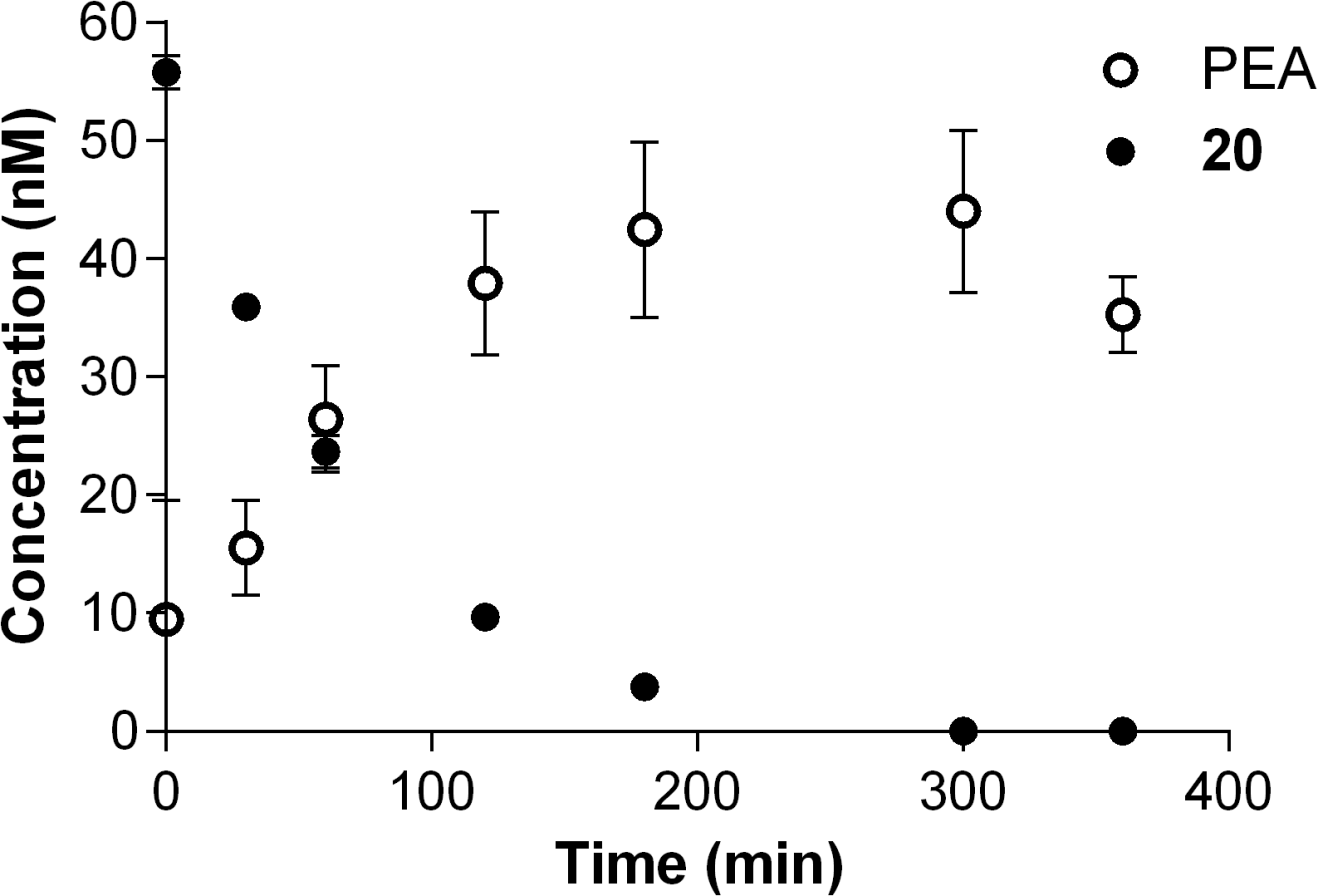
L-Val-PEA (20) biotransformation and PEA release in rat plasma. Time course of L-Val-PEA (20, ●) hydrolysis in 80% v/v rat plasma and corresponding release of PEA (1,○).

We thus focused our attention on amino acid ester-based prodrugs, modulating the physicochemical properties of the amino acid pro-moiety to get information on structure-stability relationships. The increasing steric bulk of the hydrophobic pro-moieties L-Ala- (**19**), L-Val- (**20**) and L-Ile-PEA (**21**) led to a parallel reduction in rates of conversion to **1** in both biological matrices. In fact, while L-Ala-PEA was quickly hydrolyzed both in plasma and liver homogenate (t_1/2_ ~ 1 min), introduction of a branched alkyl side chain in L-Val and L-Ile derivatives led to a 46- and 200-fold increase in plasma half-life, respectively. The behavior of L-Val-PEA (**20**) in rat plasma was of particular interest. As shown in Fig 7, the time course of its hydrolysis showed a gradual conversion into PEA, with maximal release after 5 h (C_max_ = 44 nm). This behavior was considered promising for the aim to sustain PEA plasma and tissue levels.

A similar trend in prodrug stability was also observed in rat liver homogenate, where an increased steric bulk around the ester moiety led to lower bioconversion, with 65% of unmodified compound after 2 h of incubation for L-Ile-PEA (**21**). L-Val-PEA showed improved stability in rat liver homogenates, compared, to the L-Ala analog, even if its half-life (26 min) was similar to that of PEA itself, thus not excluding degradation of the active constituent. L-Trp-PEA (**22**) with a large, planar and hydrophobic amino acid side chain was rapidly converted in rat plasma (t_1/2_ ~ 5 min) and, in rat liver, its half-life was only slightly longer (t_1/2_ ~ 36 min) than that of L-Val-PEA (**20**). We also explored the effect of the hydrophilic amino acids L-Asn (**23**) and L-Gln (**24**). Compound **24** was more stable than its shorter homologue **23** in both biological matrices. In rat plasma, **23** released PEA quickly with a *t*_1/2_ ~ 9 min, as a result of the low chemical stability of the prodrug (*t*_1/2_ ~ 3.3 min in buffered solution at pH 7.4). Compound **24** showed higher stability, particularly in rat liver (*t*_1/2_ ~ 80 min), suggesting that specific recognition events affect compound degradation in rat liver homogenate. PEA was conjugated to L-Asp via either the α- (L-Asp-α-PEA, **26**) or the ω- (L-Asp-ω-PEA, **29**) carboxylic group. The introduction of an ionizable acidic moiety led to a marked increase in rat plasma stability with half-lives ranging from 172 min (**26**) to 262 min (**29**), resulting in a release of PEA with a maximal concentration (C_max_) of about 26 and 34 nm at 4 h and 6 h, respectively. In rat liver, the two pro-moieties gave different results, with L-Asp-ω-PEA (**29**) being comparable to L-Val-PEA (**20**), while L-Asp-α-PEA (**26**) was one of the most stable derivatives (66% of compound left after 2 h). The introduction of a zwitterionic amino acid moiety could therefore help in increasing both rat plasma and rat liver stability.

Finally, the effect of inversion of the chiral center was explored by introducing two amino acids belonging to the D-series, D-Asn (**27**) and D-Val (**28**). Rat plasma stability of D-Asn was low and comparable to that of its L stereoisomer. This result can be influenced by the low chemical stability observed for **27**. In rat liver, D-Asn-PEA was more stable than L-Asn-PEA, with a half-life of 55 min. The chiral inversion in Val-PEA prodrugs had a marked effect on stability in both biological matrices. In fact, a plasma half-life of 173 min was obtained for **28**, over 6-fold that of L-Val-PEA, but with a lower conversion to PEA (C_max_ = 20 nm). D-Val-PEA (**28**) was much more stable in rat liver as well, as 67% of prodrug was still present after 2 h of incubation. These results, particularly in the case of Val-PEA prodrugs, showed that inversion of chirality could effectively increase stability towards plasma and liver hydrolases.

**Fig 8:**
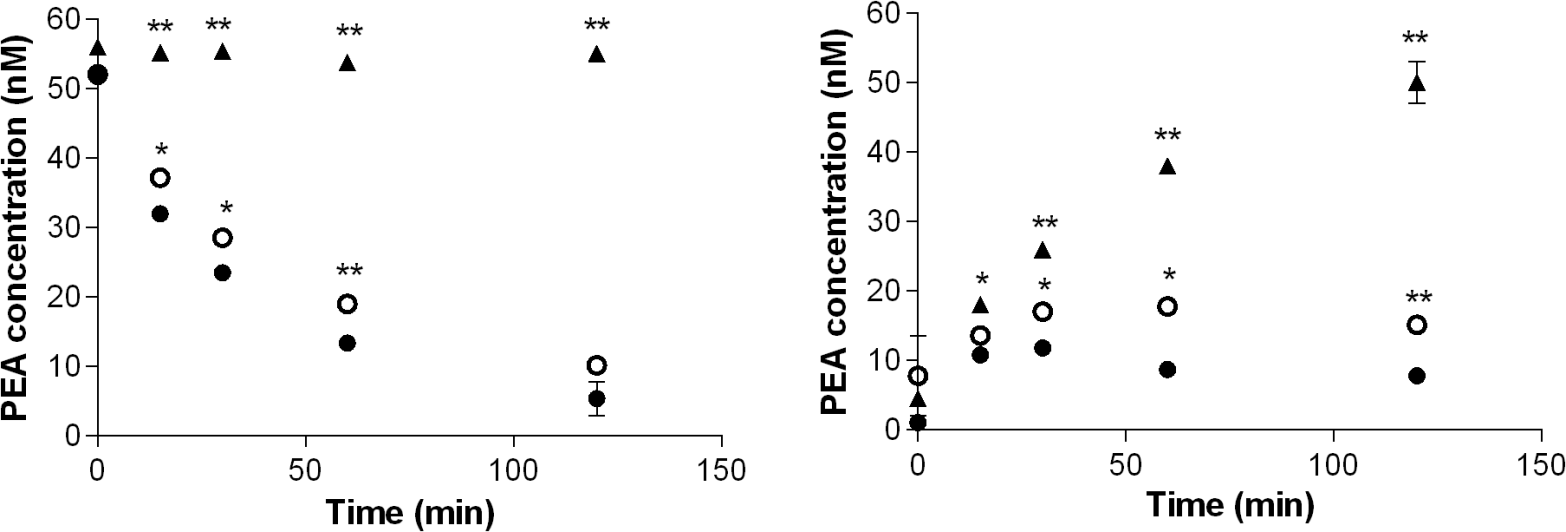
Role of amidase activity on compound degradation in rat liver homogenate. PEA levels in rat liver homogenate after addition of 50 nm PEA (left) or 50 nm L-Val-PEA prodrug (right) and pre-treatment with FAAH inhibitor URB597 at the final concentration of 100 nm (○) and 10 μm (▲), if compared to controls (●); data are expressed as means ± SEM, n = 3. * *P*<0.05; ** *P*<0.005.

Prodrug transformation in rat liver homogenate did not result in a quantitative release of PEA, the C_max_ of which being lower than 30 nm for all tested prodrugs (see Figs E-Q in S2 File). This is likely due to the occurrence of degradative reactions in this assay, that presumably not only hydrolyzed the ester group, but also the amide function. This is consistent with the strong amidase activity of rat liver homogenate observed on PEA, and with the known high expression of FAAH in rat liver. To test the importance of amidase activity on prodrug degradation in liver homogenate, PEA and the prodrug L-Val-PEA (**20**) were incubated after pretreatment (10 min) with the FAAH inhibitor URB597 (Fig 1), which has an IC_50_ of about 3 nm on liver FAAH [47]. At the concentration of 100 nm URB597 caused partial, but statistically significant, increase of PEA concentrations if compared to control incubations of both PEA and **20** (Fig 8, p<0.05). Pre-incubation with a higher concentration of URB597 (10 μm) led to complete inhibition of PEA hydrolysis and to a quantitative conversion of **20** into PEA. At this higher concentration URB597 likely loses its selectivity, targeting other serine hydrolases in rat liver, such as carboxylesterases [48,49]. We conclude therefore that in rat liver homogenate both PEA and **20** are substrates of FAAH, as well as of other uncharacterized enzymes with amidase activity versus PEA.

To further evaluate the role of amide bond hydrolysis in prodrug transformation, we prepared and tested (*S*)- and (*R*)-α-methyl-PEA (**30** and **31**). These compounds are characterized by the presence of a methyl group on the ethanolamine α-carbon which sterically hampers enzymatic hydrolysis of the amide bond. Similarly, the analogs (*S*)- and (*R*)- α-methyl-OEA had been shown to be stable in mouse liver homogenate and the plasma and liver *C*_max_ of (*R*)-α-methyl-OEA were significantly higher than those of OEA when administered to rats by the oral route [50]. As expected, compounds **30** and **31** were stable in both phosphate buffer and rat plasma. They were stable in rat liver homogenate as well, with 79% and 87% of compound unaltered after 2 h, respectively (Figs. R-S in S2 File). We therefore prepared the L-Val ester prodrug of (*R*)-α-methyl-PEA to compare its stability with that of the L-Val prodrug of PEA (**20**). The L-Val-(*R*)-α-methyl-PEA prodrug **33** was stable in phosphate buffer and its half-life in rat plasma (190 min) was 4-fold longer than that of **20**. In rat liver homogenate compound **33** showed a significantly longer half-life (38 min versus 26 min for **20**) and, interestingly, a greater conversion to the parent (*R*)-α-methyl-PEA (C_max_ of released PEA = 29 nM, see Fig T in S2 File, versus 12 nm for **20**). This result underlines that, in rat liver homogenate, hydrolysis of the amide bond in the PEA moiety of **20** and other amino acid esters exerts a significant role in prodrug degradation and could represent a limitation to the release of PEA *in vivo* after oral administration of these prodrugs.

**Fig 9:**
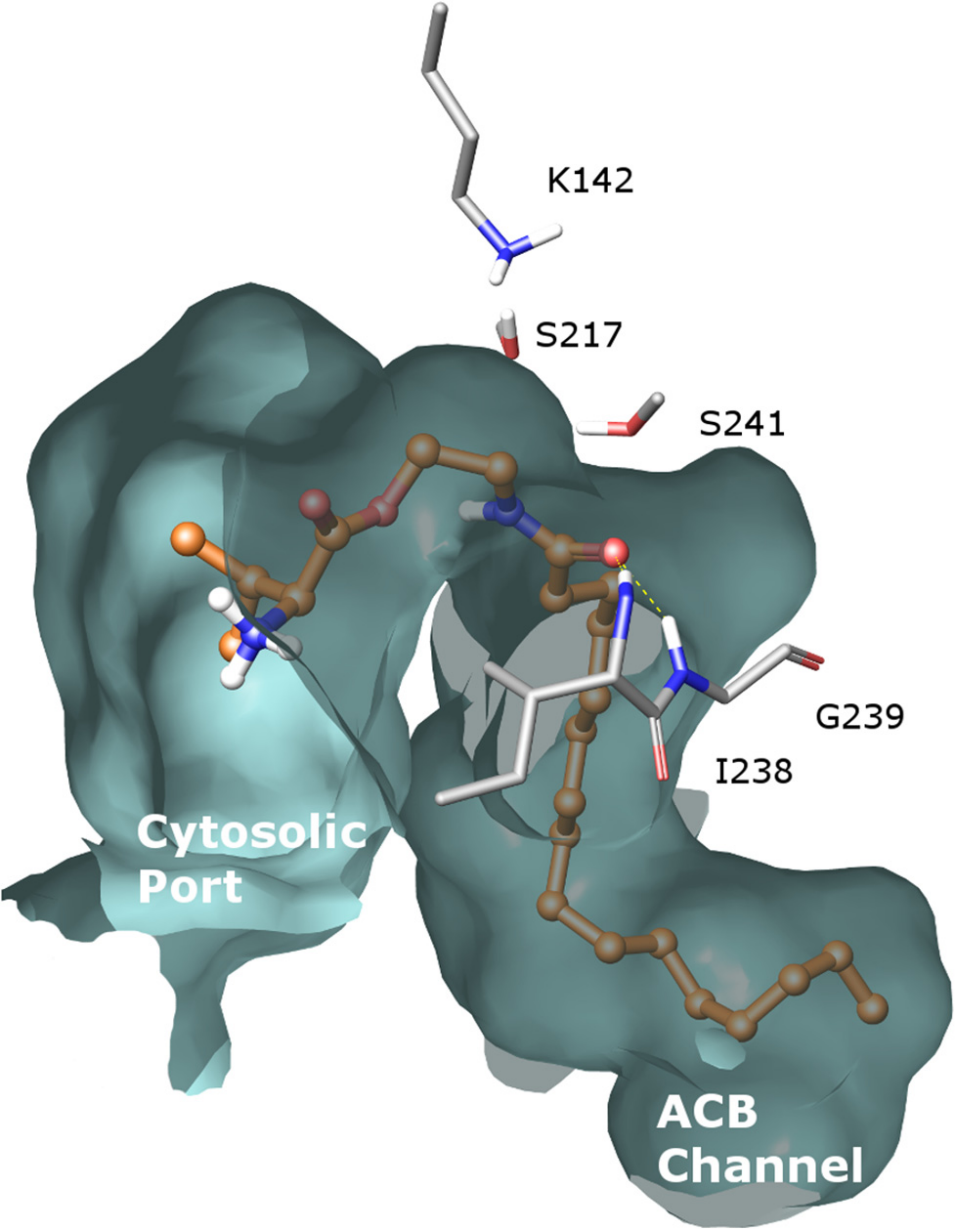
Docking of L-Val-PEA (20) into rat FAAH active site. Representation of L-Val-PEA (**20**) docked into rat FAAH active site. The amide group undertakes hydrogen bonds with the two NH groups of I238 and G239 belonging to the oxyanion hole and with the backbone carbonyl of M191 (not shown). The catalytic triad S217, S241 and K142 is also displayed. The palmitoyl portion fits the acyl chain binding (ACB) channel and the valine occupies the cytosolic port.

The possibility that L-Val-PEA (**20**) can be a substrate of endogenous amidases is also supported by docking studies into rat FAAH crystal structure. Considering the poses of compound **20** with the amide group of PEA close to the catalytic serine 241 and its carbonyl oxygen interacting with the NH groups in the oxyanion hole, the palmitoyl portion fits the acyl chain binding pocket, which is occupied, in a crystal structure, by the hydrophobic chain of the FAAH inhibitor MAFP [38]. The valine pro-moiety can be easily accommodated within a large pocket, delimited by a hydrophilic region, also referred to as the cytosolic port, at the interface between the two FAAH monomers of the dimeric structure (Fig 9). This pose is consistent with the possibility that amino acid derivatives of PEA are substrates of FAAH in liver, as well as in other tissues. Of course, this does not exclude that these prodrugs could also be substrates of other amidases.

### *In vivo* assessment of valine prodrugs in Wistar rats

**Fig 10:**
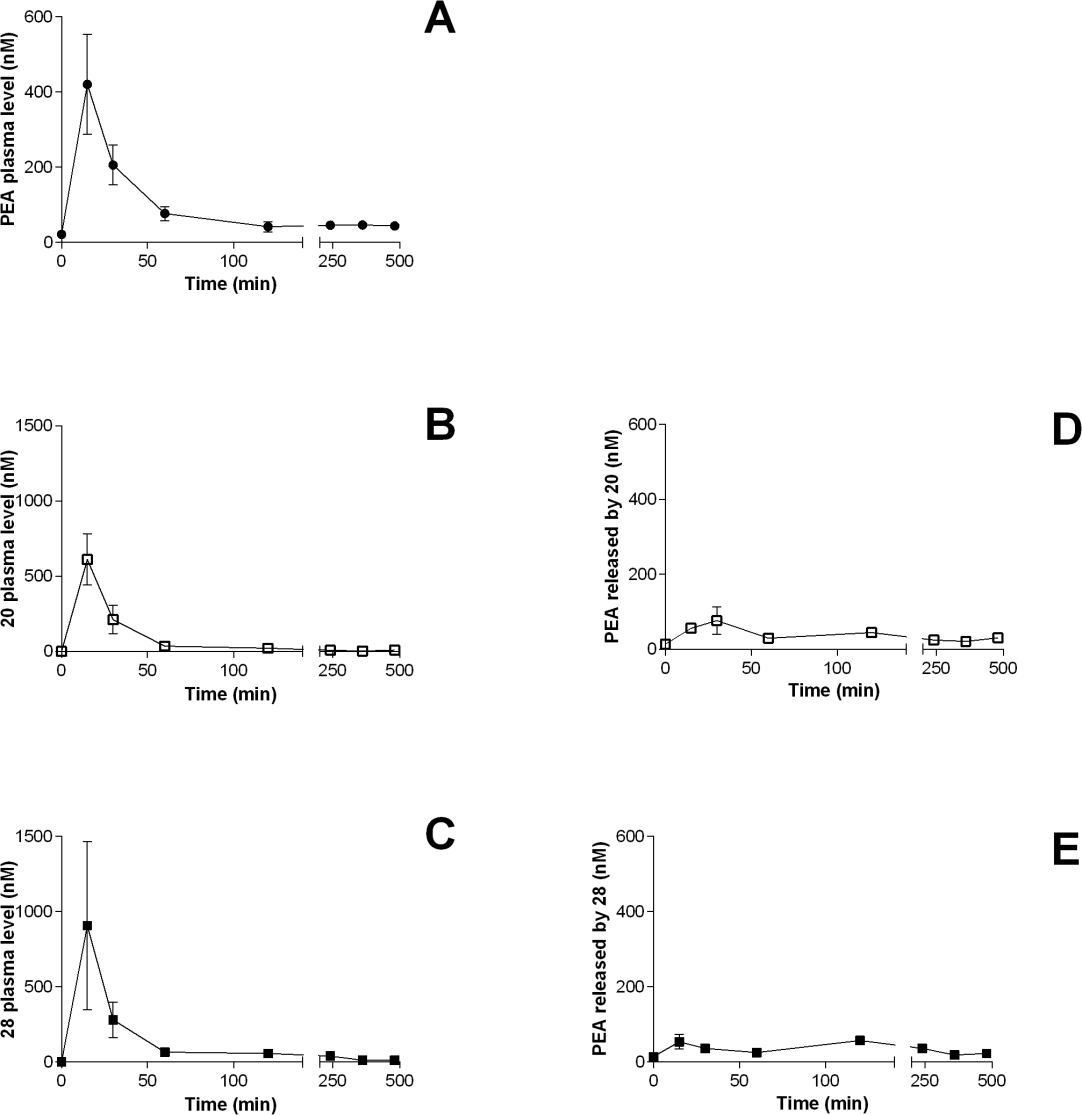
Prodrug and PEA plasma levels after oral administration to rats. a-c) Plasma levels of PEA (●), **20** (□) and **28** (■) after a single oral administration to male Wistar rats of equimolar doses (PEA: 100 mg kg^-1^; **20** and **28**: 145 mg kg^-1^). d-e.) Plasma levels of released PEA after a single oral administration of **20** (□) and **28** (■). Data are expressed as means ± SEM, n = 9.

The prodrug L-Val-PEA (**20**) showed a promising conversion rate to PEA in rat plasma, while its enantiomer D-Val-PEA (**28**) showed reduced hepatic clearance. As these are the two key factors regulating the efficiency of PEA release *in vivo*, both **20** and **28** were submitted to oral exposure studies in Wistar rats and compared to PEA (**1**). Compounds were orally administered at the equimolar doses of 100 mg kg^-1^ (PEA) and 145 mg kg^-1^ (**20**, **28**), and rat plasma concentrations were measured at 15 min, 30 min and 1, 2, 4, 6 and 8 h time points (Table 2 and Fig 10).

**Table 2:** Plasma concentrations (nM) after p.o. administration of PEA (1), 20 and 28.^a^

	1	20		28	
Time (min)	PEA Conc	20 Conc	PEA Conc	28 Conc	PEA Conc
0	21.3 (± 3.7)	-	13.5 (± 0.7)	-	14.0 (± 0.4)
15	420.1 (± 132.4)	611.6 (± 169.7)	56.4 (± 13.5)	906.6 (± 559.8)	53.9 (± 19.7)
30	205.9 (± 53.0)	212.0 (± 94.5)	76.8 (± 36.3)	280.3 (± 118.4)	36.3 (± 9.4)
60	76.6 (± 18.7)	35.8 (± 11.5)	29.7 (± 4.8)	65.9 (± 19.8)	25.2 (± 4.1)
120	41.9 (± 13.5)	22.2 (± 6.1)	44.9 (± 10.0)	56.4 (± 9.3)	57.4 (± 8.5)
240	45.8 (± 7.9)	7.1 (± 2.5)	24.5 (± 2.6)	39.9 (± 17.9)	36.4 (± 10.2)
360	46.5 (± 10.5)	2.7 (± 0.9)	21.0 (± 1.6)	11.4 (± 4.3)	18.6 (± 2.0)
480	43.6 (± 7.0)	9.3 (± 4.6)	30.8 (± 6.9)	10.1 (± 2.0)	23.0 (± 2.8)

^a^ Values are expressed as the mean ± SEM, n = 9.

Upon oral administration of PEA, the highest plasma concentration was observed after 15 min (C_max_ = 420 ± 132 nm) corresponding to a 20-fold increase of its basal values (21 ± 4 nM). Similarly to what had been observed in dogs [14], PEA plasma levels dropped 2 h after administration to concentrations very close to the basal ones.

L-Val- (**20**) and D-Val-PEA (**28**) prodrugs reached their C_max_ in rat plasma 15 min after administration. For L-Val-PEA the mean C_max_ value was 612 nm, while for D-Val-PEA a higher mean value was obtained (907 nm), although this difference was not statistically significant (p>0.05). AUC_0-__480_ was 7728(±1986) and 13836(±2886) ng min/mL (mean±SEM, n = 9) for L-Val- and D-Val-PEA, respectively, while it was 6525(±1372) ng min/mL for PEA. These data indicate that, particularly in the case of D-Val-PEA (**28**), the prodrugs are slightly more available than PEA. This can be attributed to their higher solubility due to the presence of a basic group and/or, at least for **28**, increased resistance to amidase-mediated hydrolysis in the liver. The concentrations of PEA released after administration of compounds **20** and **28** are reported in Table 2. At t = 15 min levels of PEA were about 54–56 nm, 8-fold lower than those obtained at the same time after oral administration of PEA, regardless of the inverted chirality of the amino acid pro-moiety. Levels of PEA released by both prodrugs at longer times were comparable to those obtained by direct administration of equimolar PEA (Table 2).

L-Val- and D-Val-PEA did not behave as reservoirs progressively releasing PEA into plasma. In fact, prodrug disappearance from plasma was not accompanied by a corresponding increase of PEA levels. The explanation of this behavior is probably multi-factorial. A first aspect to consider is the observed lack of *in vitro*/*in vivo* correlation. We tested two prodrugs with promising profiles in terms of conversion rate to PEA in plasma (**20**) or of reduced hepatic clearance (**28**). Given the *in vivo* results, we may conclude that **20** was too labile in the liver to be efficiently converted into PEA in plasma. For compound **28,** more stable in the liver and more bioavailable than **20** and PEA, its very long plasma half-life could have favored its degradation *in vivo*, rather than its conversion into PEA. In fact, comparing plasma levels of **20** and **28** at 15 and 30 min, while concentrations of both prodrugs showed a marked decrease, plasma levels of PEA in rats treated with **20**, but not **28**, increased. This difference may be due to the lower propensity of **28** to be converted to PEA by plasma esterases. The lack of PEA appearance in plasma could also be due to rapid distribution to lipophilic tissues where the prodrugs can be stored and/or cleaved. In fact, the increase of PEA levels at 120 min, when prodrug levels in plasma had already decreased, could be the consequence of redistribution from some tissue storage.

We observed high variability in plasma levels. For PEA, it can be explained by its unfavorable physicochemical properties, particularly its high lipophilicity and poor solubility, which make absorption through the gastro-intestinal tract extremely variable. The prodrugs have rather improved physicochemical properties, as revealed by their higher systemic exposure, but their plasma levels presented high inter-individual variability as well. This suggests that chemical derivatization of PEA needs to be supplemented with accurate formulation studies.

Further chemical work is needed to reduce amide instability, e.g. in the liver, for the prodrugs, in order to improve the ratio between activation and degradation. As also suggested by docking of a prodrug into FAAH catalytic site, an amino acid linked at the PEA hydroxyl group is not sufficient to avoid accommodation of the prodrug within the catalytic pocket of this enzyme. On the other hand, resistance to hydrolysis in the liver must be complemented by the ability to release PEA in plasma at appropriate rate.

## Conclusions

Looking for a prodrug candidate of palmitoylethanolamide (PEA), nineteen carbonate, carbamate and ester derivatives were synthesized and tested for their conversion into PEA in rat plasma and liver homogenate. *In vitro* data suggest that amino acid esters of PEA could be regarded as the most promising class of prodrugs among those here prepared and tested. Two candidate prodrugs of PEA (**20** and **28**) were tested *in vivo* to assess their systemic bioavailability and PEA exposure by the oral route. Both prodrugs showed some improvements in their bioavailability, compared to PEA, but they failed to achieve an efficient release of PEA into plasma.

While other issues (formulation, distribution) should be considered for a final evaluation of this class of prodrugs, we think these results point out that further efforts combining the synthesis of new bio-reversible PEA derivatives and their screening by *in vitro* tests with animal plasma and liver homogenate could afford a better balance between PEA release by plasma esterases and prodrug degradation by tissue amidases. Combined with the assessment of *in vivo* pharmacokinetics, this could result in the development of PEA prodrugs with improved pharmacokinetic and therapeutic properties.

## Supporting Information

S1 File MRM transitions. (PDF)

S2 File Time courses for biotransformation of PEA and its prodrugs. (PDF)
