# Supplementary material for: Amino Acid Derivatives as Palmitoylethanolamide Prodrugs: Synthesis, In Vitro Metabolism and In Vivo Plasma Profile in Rats
Source: PLoS One. 2015 Jun 8;10(6):e0128699. doi: 10.1371/journal.pone.0128699 (PMC4460047; doi:10.1371/journal.pone.0128699)
Supplement: S1 File — (PDF) [file pone.0128699.s001.pdf]

S1 File. MRM transitions. Transitions monitored for compounds 3-5, 7-9, 19-24, 26-33 in HPLC-ESI-MS/MS analysis with corresponding tube lens voltages and collision energies reported in parentheses.

3: m/z 416.3 → m/z 282.1 + m/z 344.3 (tube lens 74 V; collision energy 19, 7 eV); **4**: m/z 444.4 → m/z 106.2 + m/z 282.3 + m/z 344.3 (tube lens 74 V; collision energy 22, 19, 8 eV); **5**: m/z 484.4 → m/z 106.1 + m/z 282.3 + m/z 344.4 (tube lens 72 V; collision energy 25, 21, 13 eV); 7: m/z 399.3 → m/z 57.4 + m/z 71.3 + m/z 163.2+ m/z 282.3 (tube lens 62 V; collision energy 37, 33, 14, 13 eV); **8**: m/z 415.3 → m/z 282.2 (tube lens 66 V; collision energy 11 eV); **9**: m/z 443.3 → m/z 282.3 + m/z 300.3 + m/z 302.3 (tube lens 70 V; collision energy 12, 13, 67 eV); **19**: m/z 371.3 → m/z 57.3 + m/z 282.2 + m/z 283.2 + m/z 300.3 (tube lens 66 V; collision energy 35, 13, 16, 11 eV); **20**: m/z 399.3 → m/z 72.2+m/z 282.2 + m/z 300.2 (tube lens 105 V; collision energy 20, 15, 12 eV); **21**: m/z 413.4 → m/z 86.2 + m/z 282.3 + m/z 300.3 (tube lens 73 V; collision energy 18, 15, 13 eV); **22**: m/z 486.4 → m/z 132.1 + m/z 159.1 + m/z 282.2 (tube lens 85 V; collision energy 36, 23, 18 eV); **23**: m/z 414.3 → m/z 57.3 + m/z 282.2 + m/z 300.2 (tube lens 72 V; collision energy 46, 19, 13 eV); **24**: m/z 428.4 → m/z 130.1 + m/z 147.1 + m/z 282.3 (tube lens 75 V; collision energy 22, 12, 16 eV); **26**: m/z 415.2 → m/z 119.3 + m/z 282.3 + m/z 383.3 (tube lens 74 V; collision energy 26, 17, 14 eV); **27**: m/z 414.4 → m/z 57.4 + m/z 282.3 + m/z 300.3 (tube lens 61 V; collision energy 43, 16, 10 eV); **28**: m/z 399.4 → m/z 72.3 + m/z 282.3 + m/z 300.3 (tube lens 65 V; collision energy 15, 12, 12 eV); **29**: m/z 415.3 → m/z 119.3 + m/z 282.3 + m/z 284.4 (tube lens 79 V; collision energy 15, 12, 25 eV); **30**: m/z 314.3 → m/z 76.3+ m/z 58.4 (tube lens 71 V; collision energy 14, 17 eV); **31**: m/z 314.3 → m/z 76.3 + m/z 58.4 (tube lens 71 V; collision energy 14, 17 eV); **33**: m/z 413.6 → m/z 296.3 + m/z 314.3 (tube lens 68 V; collision energy 14, 11 eV).
