# Supplementary material for: Amino Acid Derivatives as Palmitoylethanolamide Prodrugs: Synthesis, In Vitro Metabolism and In Vivo Plasma Profile in Rats
Source: PLoS One. 2015 Jun 8;10(6):e0128699. doi: 10.1371/journal.pone.0128699 (PMC4460047; doi:10.1371/journal.pone.0128699)
Supplement: S2 File — (PDF) [file pone.0128699.s002.pdf]

S2 File. Time courses for biotransformation of PEA and its prodrugs. Time courses of PEA or prodrug biotransformation and corresponding release of PEA in 80% v/v rat plasma and in rat liver homogenate.

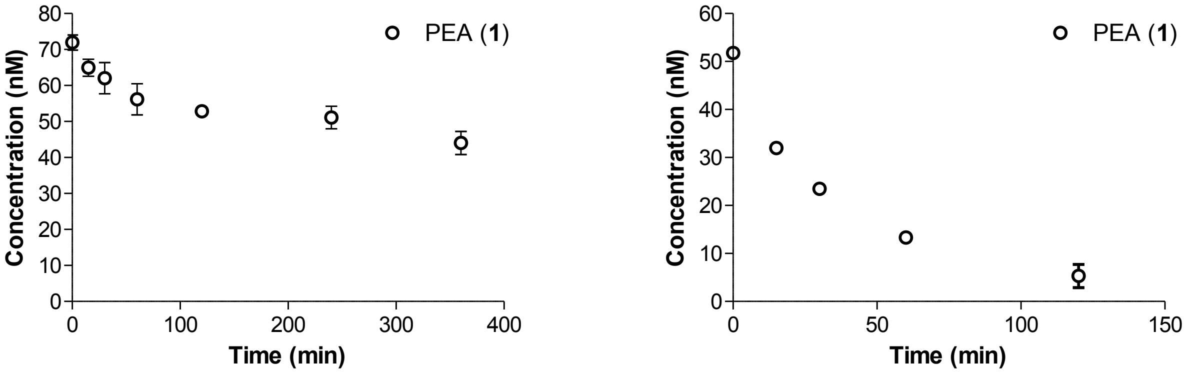

Fig. A. Time course of PEA levels in 80% v/v rat plasma (left graph) and liver homogenate (right graph).

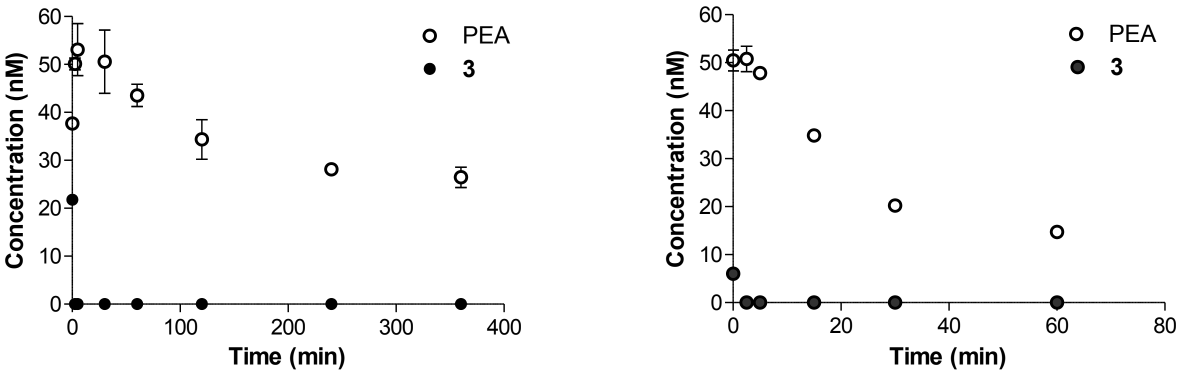

Fig. B. Time course of **3** and PEA levels in 80% v/v rat plasma (left graph) and liver homogenate (right graph).

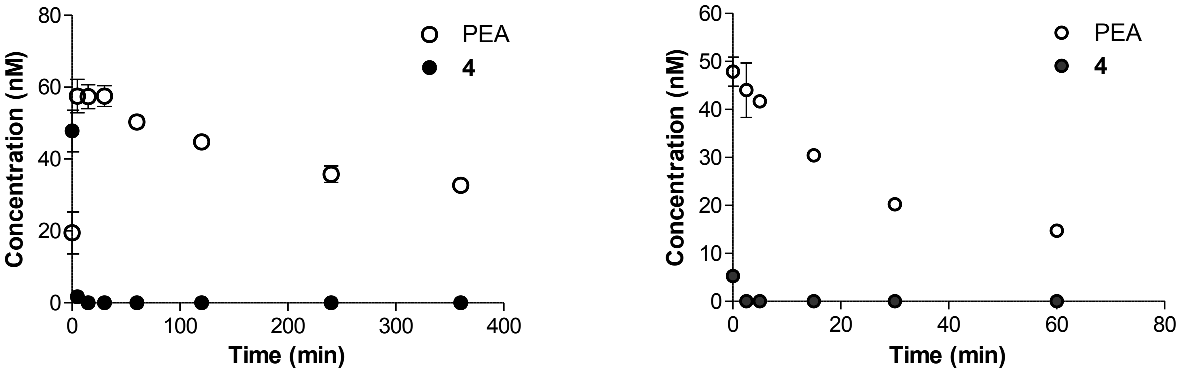

Fig. C. Time course of **4** and PEA levels in 80% v/v rat plasma (left graph) and liver homogenate (right graph).

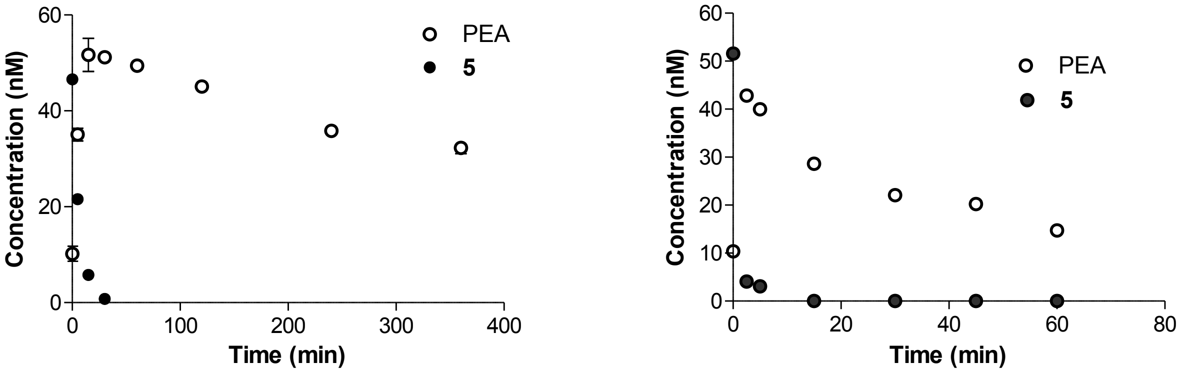

Fig. D. Time course of **5** and PEA levels in 80% v/v rat plasma (left graph) and liver homogenate (right graph).

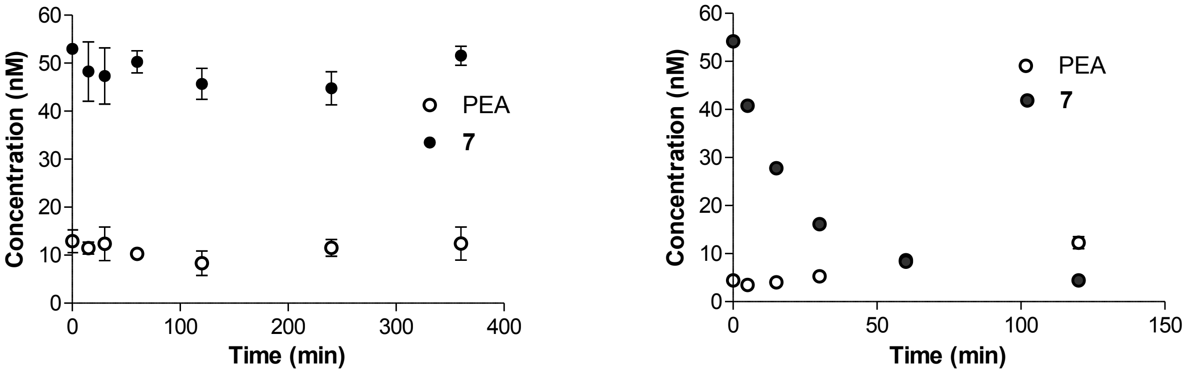

Fig. E. Time course of **7** and PEA levels in 80% v/v rat plasma (left graph) and liver homogenate (right graph).

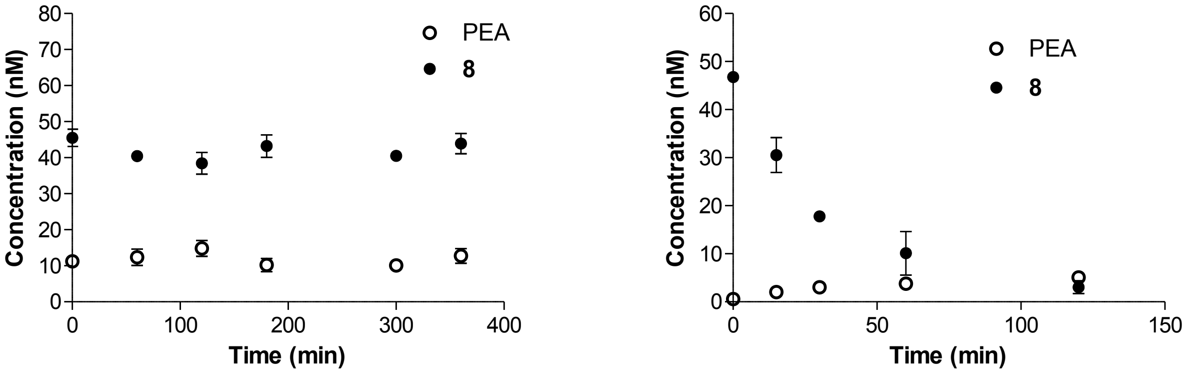

Fig. F. Time course of **8** and PEA levels in 80% v/v rat plasma (left graph) and liver homogenate (right graph).

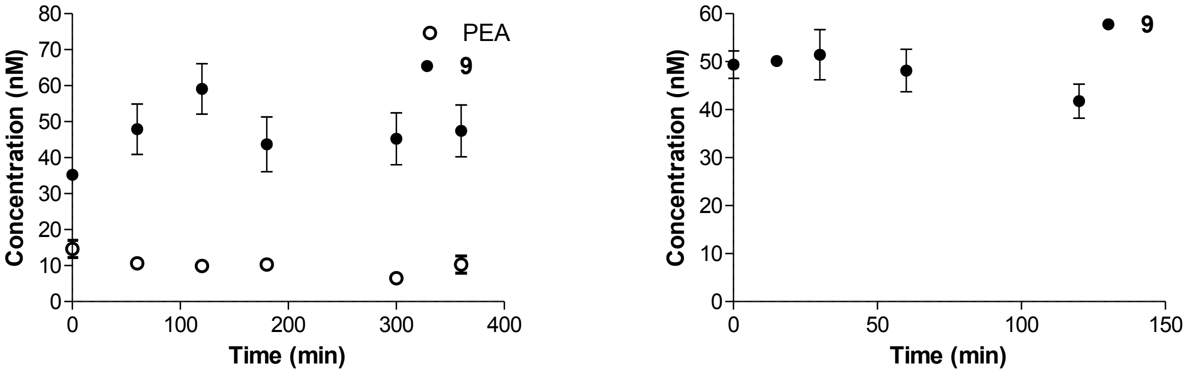

Fig. G. Time course of **9** and PEA levels in 80% v/v rat plasma (left graph) and liver homogenate (right graph).

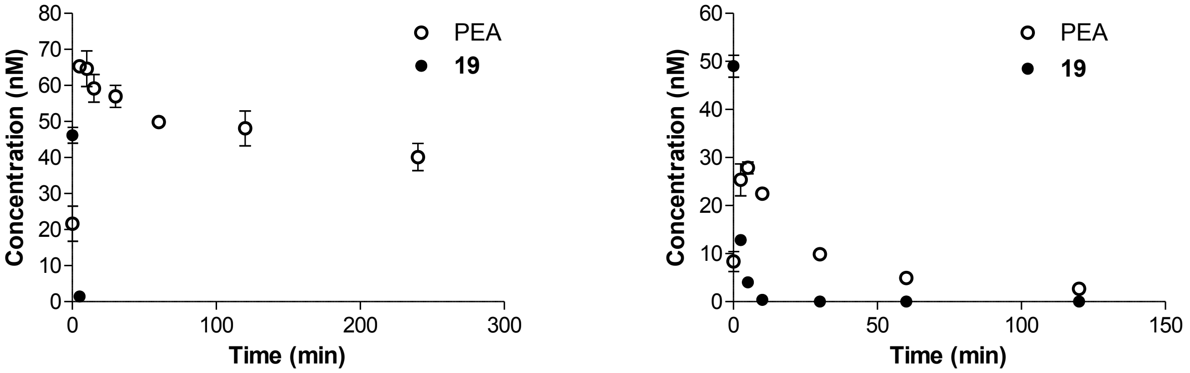

Fig. H. Time course of **19** and PEA levels in 80% v/v rat plasma (left graph) and liver homogenate (right graph).

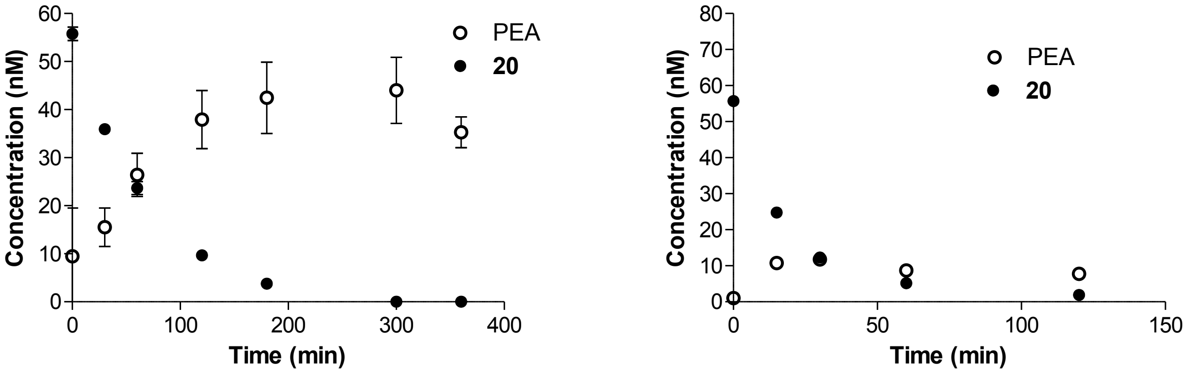

Fig. I. Time course of **20** and PEA levels in 80% v/v rat plasma (left graph) and liver homogenate (right graph).

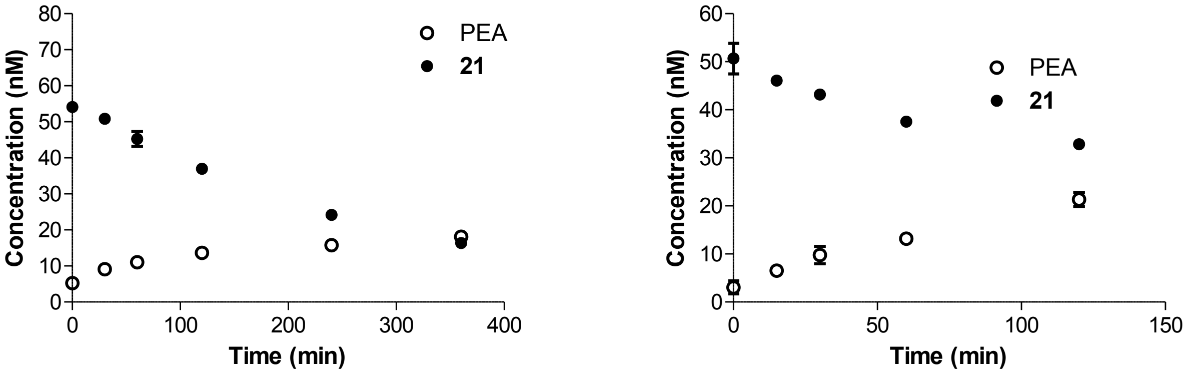

Fig. J. Time course of **21** and PEA levels in 80% v/v rat plasma (left graph) and liver homogenate (right graph).

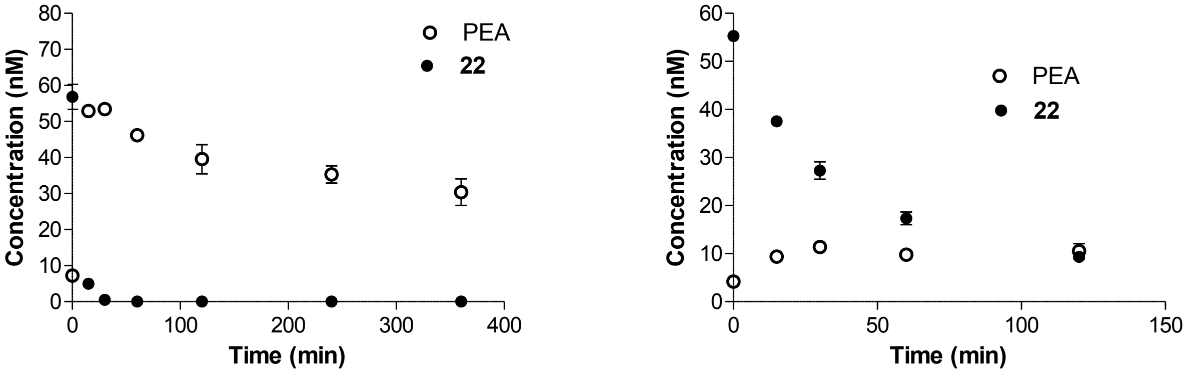

Fig. K. Time course of **22** and PEA levels in 80% v/v rat plasma (left graph) and liver homogenate (right graph).

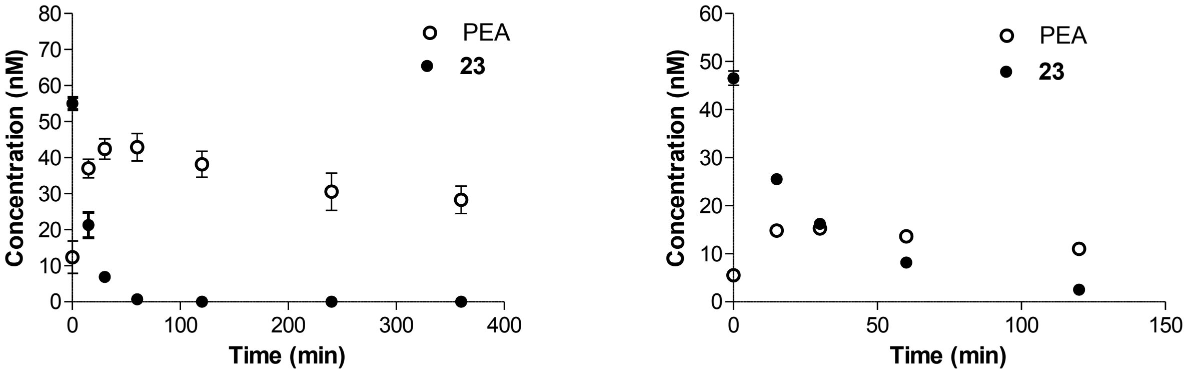

Fig. L. Time course of **23** and PEA levels in 80% v/v rat plasma (left graph) and liver homogenate (right graph).

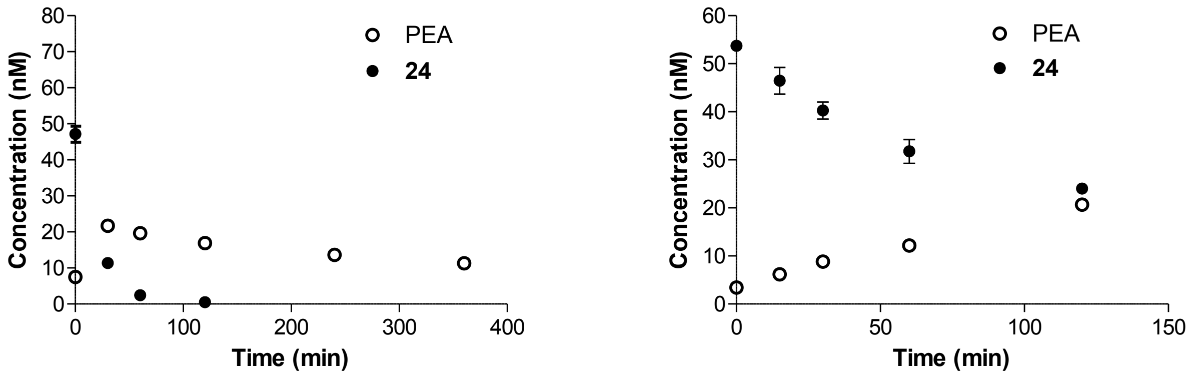

Fig. M. Time course of **24** and PEA levels in 80% v/v rat plasma (left graph) and liver homogenate (right graph).

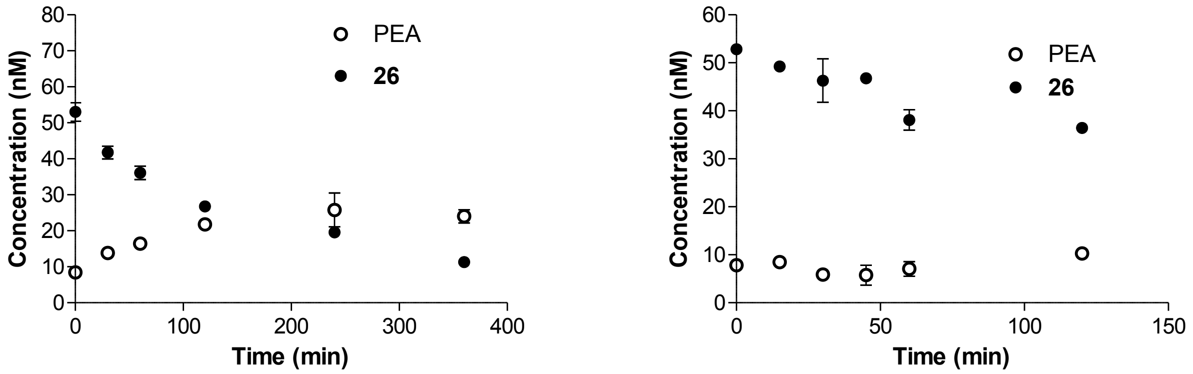

Fig. N. Time course of **26** and PEA levels in 80% v/v rat plasma (left graph) and liver homogenate (right graph).

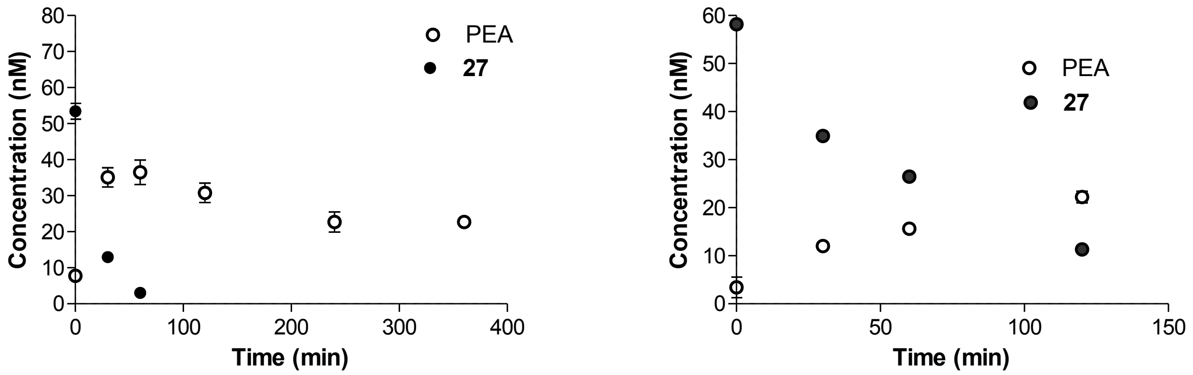

Fig. O. Time course of **27** and PEA levels in 80% v/v rat plasma (left graph) and liver homogenate (right graph).

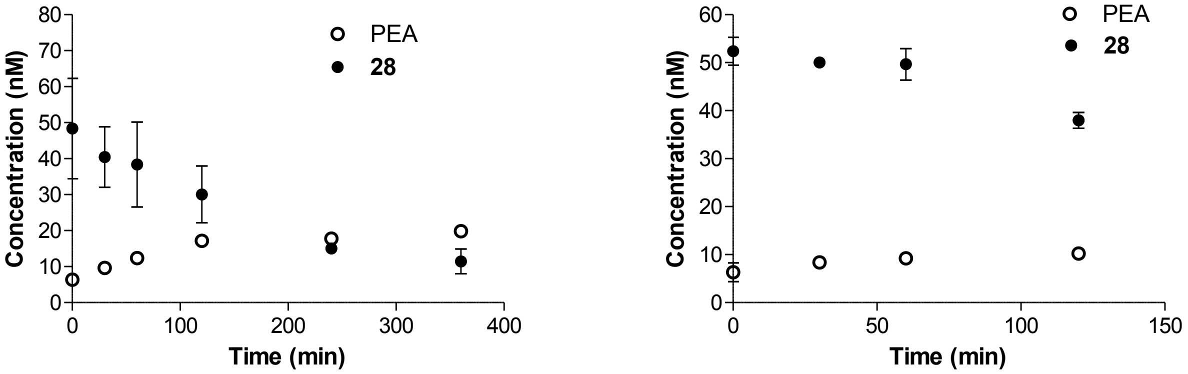

Fig. P. Time course of **28** and PEA levels in 80% v/v rat plasma (left graph) and liver homogenate (right graph).

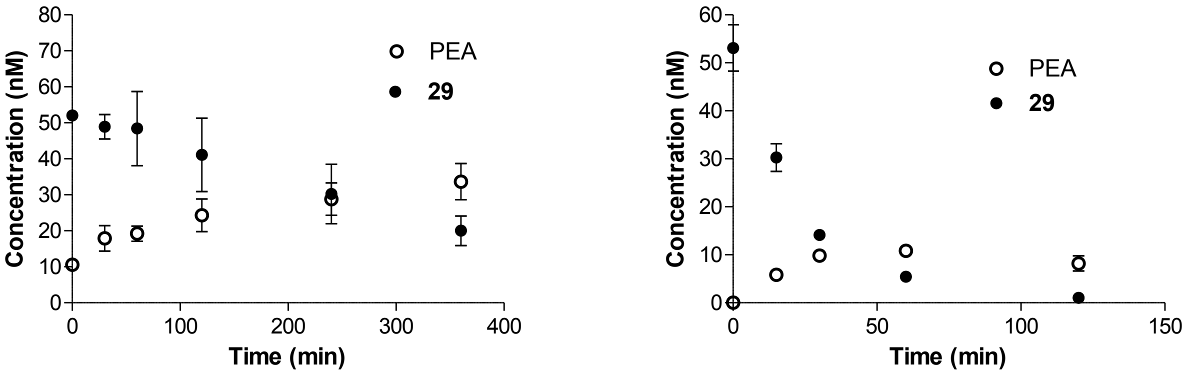

Fig. Q. Time course of **29** and PEA levels in 80% v/v rat plasma (left graph) and liver homogenate (right graph).

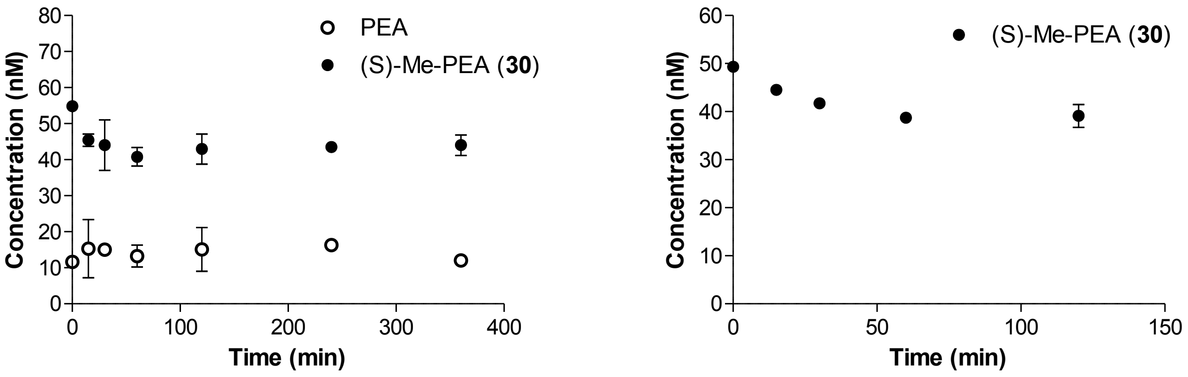

Fig. R. Time course of **30** levels in 80% v/v rat plasma (left graph) and liver homogenate (right graph).

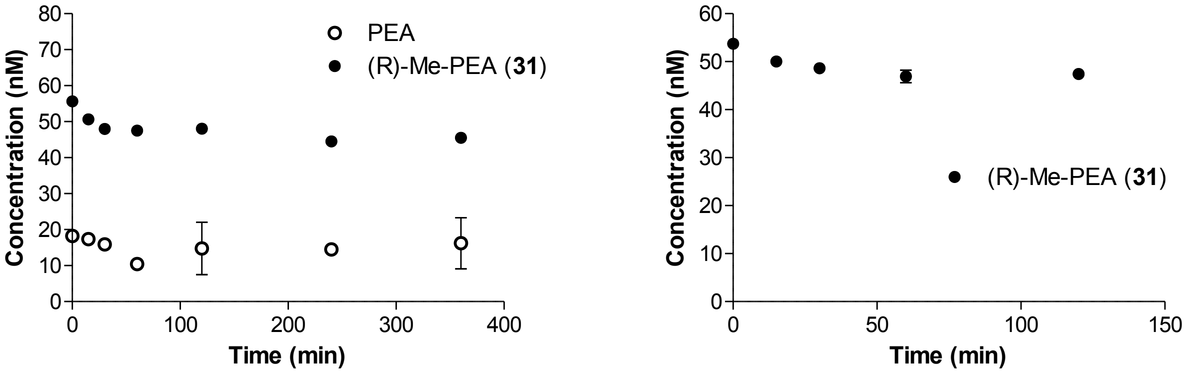

Fig. S. Time course of **31** levels in 80% v/v rat plasma (left graph) and liver homogenate (right graph).

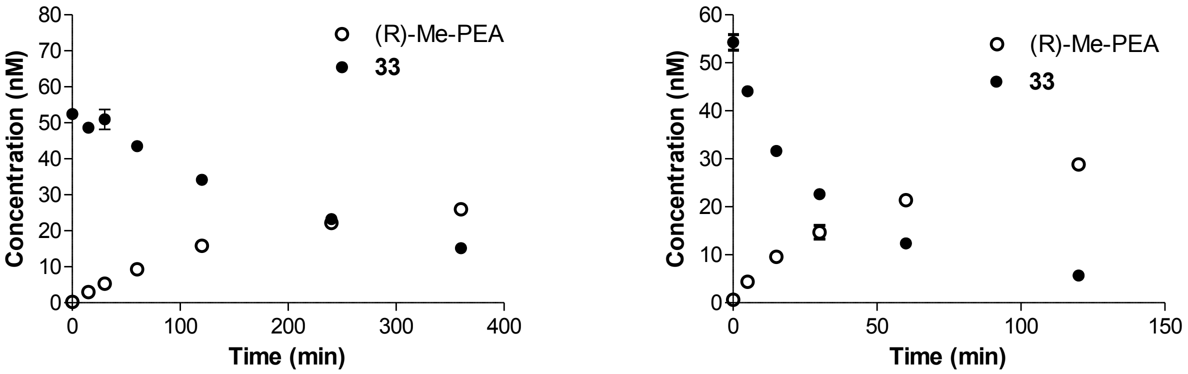

Fig. T. Time course of **33** and (*R*)-methyl-PEA levels in 80% v/v rat plasma (left graph) and liver homogenate (right graph).
